# Understanding the Enablers and Barriers to Appropriate Infants and Young Child Feeding Practices in India: A Systematic Review

**DOI:** 10.3390/nu13030825

**Published:** 2021-03-02

**Authors:** Mansi Vijaybhai Dhami, Felix Akpojene Ogbo, Blessing Jaka Akombi-Inyang, Raphael Torome, Kingsley Emwinyore Agho, on behalf of the Global Maternal and Child Health Research Collaboration (GloMACH)

**Affiliations:** 1 Translational Health Research Institute (THRI), Western Sydney University, Penrith, NSW 2571, Australia; f.ogbo@westernsydney.edu.au (F.A.O.); k.agho@westernsydney.edu.au (K.E.A.); 2 Barmera Medical Clinic (Lake Bonney Private Medical Clinic), Barmera, SA 5345, Australia; rtorome@barmeramedical.com.au; 3 School of Population Health, University of New South Wales, Sydney, NSW 2052, Australia; b.akombi@unsw.edu.au; 4 School of Social Sciences, Western Sydney University, Penrith, NSW 2571, Australia; 5 School of Health Sciences, Western Sydney University, Penrith, NSW 2571, Australia; 6 African Vision Research Institute (AVRI), University of KwaZulu-Natal, Durban 4041, South Africa

**Keywords:** infant and young child feeding, breastfeeding, complementary feeding, India

## Abstract

Despite efforts to promote infant and young child feeding (IYCF) practices, there is no collective review of evidence on IYCF enablers and barriers in India. This review was conducted using 2015 Preferred Reporting Items for Systematic reviews and Meta-Analysis (PRISMA) guidelines. Six computerized bibliographic databases, Scopus, PubMed, PsycINFO, CINAHL, Embase, and Ovid MEDLINE, were searched for published studies on factors associated with IYCF practices in India from 1 January 1993, to 30 April 2020. IYCF practices examined were early initiation of breastfeeding, exclusive breastfeeding, continued breastfeeding at one year, introduction to solid semi-solid or soft foods, minimum dietary diversity, minimum meal frequency, minimum acceptable diet, continued breastfeeding at two years, predominant breastfeeding, and bottle feeding. In total, 6968 articles were retrieved, and 46 studies met the inclusion criteria. The common enablers of IYCF were higher maternal socioeconomic status (SES) and more frequent antenatal care visits (ANC) (≥3). Common barriers to IYCF practices were low SES and less frequent ANC. The review showed that the factors associated with IYCF practices in India are largely modifiable and multi-factorial. Improving IYCF practices would require the adoption of both facilities- and community-based policy interventions at the subnational and national levels in India.

## 1. Introduction

Appropriate infant and young child feeding (IYCF, comprising of breastfeeding and complementary feeding) play important roles in optimal child growth and development. This is because appropriate breastfeeding is associated with a lower prevalence of childhood diarrhea [1,2], upper respiratory tract infection and obesity, and maternal diseases like diabetes mellitus [3]. Additionally, appropriate complementary feeding is associated with a reduced risk of undernutrition (i.e., underweight, stunting, and/or wasting) [4,5,6]. Despite the benefits of appropriate IYCF, many low- and middle-income countries (LMICs) still report higher prevalence of inappropriate IYCF [1,7,8,9,10,11,12]. In India, inappropriate IYCF practices have contributed to childhood malnutrition contributing to about 68% of the under five deaths and 83% of the neonatal deaths [13]. Inappropriate IYCF was the underlying source for an estimated 0.9 million under-five deaths in 2016 [2]. Inappropriate IYCF are feeding behaviors that do not meet the recommendations of the World Health Organization/United Nations Children’s Fund (WHO/UNICEF) indicators for assessing IYCF practices [14]. Core WHO/UNICEF recommendations include the initiation of breastfeeding for newborns within the first hour of birth, followed by exclusive breastfeeding (EBF) for the first six months of birth, and continued breastfeeding for up to two years and more, with nutritionally-balanced and safe complementary foods introduced to the infant when the child is six months old [14].

To improve global infant and young child feeding, the WHO recently endorsed a set of Global Nutrition Targets (WHO GNT, including Goals 1, 5, and 6). These goals aim to reduce the number of stunted children by 40%, increase the global EBF rate to at least 50%, and reduce and maintain childhood wasting to less than 5% by the year 2025, respectively [15]. However, there are varied reports on how many countries in LMICs are on track to meet the WHO GNT. For example, a recent study showed that only three African countries would meet the GNT for EBF [16].

In India, a recent study showed that the prevalence of EBF was 55% at the national level [17], higher than the WHO GNT for EBF. While this is commendable, improving EBF prevalence and other breastfeeding practices is also essential to further reduce the burden of diarrhea-related morbidity [2] and stunting in India [4]. Notably, recent research indicates that the prevalence of inappropriate complementary feeding is high in India [18]. The prevalence of introduction of solid, semi-solid, or soft foods (complementary foods) among infants aged 6–8 months was 42.7% nationally [19], while that of minimum dietary diversity was 22% at national level [19]. Prevalence of minimum meal frequency nationally was 35.9% [19] and minimum acceptable diet was 9.6% [19]. Inappropriate complementary feeding practices are a significant contributor to the burden of childhood underweight, stunting, and wasting in India [4,20,21].

Understanding what factors act as enablers and barriers to the broader IYCF practices across regional areas and at the national level in India is essential to improving childhood nutritional status in the country. In 2017, a systematic review conducted for India showed that complementary feeding behaviors were largely influenced by cultural practices, limited knowledge of appropriate complementary feeding practices, and low parental education [20]. While useful for informing policy interventions in India, the study has several limitations. Firstly, the study did not incorporate or assess what can influence other important IYCF behaviors (e.g., early initiation of breastfeeding (EIBF) or EBF). Secondly, the study did not capture recent studies that used recent nationally representative data in India. Hence, there is need for a comprehensive systematic review to incorporate newer IYCF indicators based on the newer health data to better guide the policymakers and public health researchers towards improving children’s health and their nutrition requirements. The availability of recent health data reflects current socioeconomic and health status of the community and suggests the need for up-to-date evidence on what can influence IYCF behaviors in the household and community. Finally, a lack of assessment of important IYCF behaviors using new data may be limited in informing multi-faceted approaches required to improve the nutritional needs of Indian children. 

Accordingly, this review aims to systematically examine published studies and report on enablers and barriers to appropriate IYCF practices in India. The evidence will help government and non-government policy decision-makers to prioritize and implement targeted interventions that aim to improve the nutritional status of Indian children.

## 2. Methods

### 2.1. Information Sources and Search Strategy

The review was conducted using the 2015 Preferred Reporting Items for Systematic Reviews and Meta-Analysis (PRISMA) guidelines provided in Supplementary Table S1 [21]. The review was registered with the International Prospective Register of Systematic Reviews (PROSPERO), and the registration number is CRD42020170021. A list of relevant MeSH words and sub-headings of keywords was generated and used to comprehensively search peer-reviewed articles from six computerized bibliographic databases: Scopus, PubMed, PsycINFO, CINAHL, Embase, and Ovid MEDLINE. The search covered research conducted in India and published between 1 January 2008, to 30 April 2020. The year 2008 was used as a baseline in this review to capture the period when the new IYCF indicators (breastfeeding and complementary feeding indicators) were introduced by the WHO [5].

The articles retrieved from each database were imported into an EndNote X8 (Clarivate Analytics, USA). We searched the bibliographical references of all retrieved articles that met the inclusion criteria, in addition to citation tracking using Google Scholar for additional relevant publications that might have been missed. The following combination of keywords was used in the search:

- (Child* or Preschool* or Pediatric* or Infant* or Bab* or Newborn* or Neonate*)
  AND
- (Feed* or Breastfeed* or Complementary Feed* or Food*)
  AND
- (Factor* or Determinant* or Correlate* or Cause* or Influence* or Enabler* or Barrier* or Promoter*)
  AND
- (India)

### 2.2. Eligibility Criteria

Studies were included in the review if they meet the following criteria: (i) focused on children under two years of age, (ii) were conducted in India, (iii) analyzed factors associated with IYCF indicators (EIBF; EBF; continued breastfeeding at one year; introduction of solid, semi-solid, or soft foods; minimum dietary diversity (MDD); minimum meal frequency (MMF); minimum acceptable diet (MAD); continued breastfeeding at two years; predominant breastfeeding; and bottle feeding), (iv) were published between 1993 and 2020, (v) were observational studies (qualitative studies, case studies, books, policy briefs, or theses were excluded), (vi) were published in a peer-reviewed journal (non-peer-reviewed research, review, or commentaries were excluded), and (vii) were written in English. Eight WHO/UNICEF IYCF indicators were selected for this review based on the available published literature at the regional and national level of India [18,22,23,24,25]. These indicators were defined using the WHO/UNICEF definitions for assessing IYCF practices [5]:

- EIBF was defined as the proportion of children born in the last 24 months who were put to the breast within one hour of birth.
- EBF was defined as the proportion of infants 0–5 months of age who receive breast milk as the only source of nourishment but are allowed oral rehydration solution, drops or syrups of vitamins, and medicines.
- Continued breastfeeding at one year was defined as the proportion of children 12–15 months of age who are fed breast milk.
- Introduction of solid, semi-solid, or soft foods was defined as the proportion of infants 6–8 months of age who receive solid, semi-solid, or soft foods.
- MDD was defined as the proportion of children 6–23 months of age who receive foods from four or more food groups. The seven foods groups used for this indicator are: grains, roots and tubers, legumes and nuts, dairy products (milk, yogurt, cheese), flesh foods (meat, fish, poultry, and liver/organ meats), eggs, vitamin-A rich fruits and vegetables, as well as other fruits and vegetables.
- MMF was defined as the proportion of breastfed and non-breastfed children 6–23 months of age who receive solid, semi-solid, or soft foods (but also including milk feeds for non-breastfed children) the minimum number of times or more (Minimum is defined as: two times for breastfed infants 6–8 months, three times for breastfed children 9–23 months, and four times for non-breastfed children 6–23 months).
- MAD was defined as the proportion of children 6–23 months of age who receive a minimum acceptable diet (apart from breast milk).
- Continued breastfeeding at two years was defined as the proportion of children 20–23 months of age who are fed breast milk.
- Predominant breastfeeding was defined as the proportion of infants 0–5 months of age who receive breast milk as the main source of nourishment but are allowed water, water-based drinks, fruit juice, oral rehydration solution, drops or syrups of vitamins, and medicines.
- Bottle feeding was defined as the proportion of children 0–23 months of age who are fed with a bottle during the previous day.

### 2.3. Data Collection Process and Data Items

All articles identified in the search were exported into EndNote X8 and used for removing duplicates, screening, and selection. A three-step screening process was then employed. In the first screening phase, the first author (MVD) screened all publications by reading the titles. The second screening phase involved reading the abstracts of studies retained from the first screening phase, and eligible articles were retained. In the final screening phase, MVD read the full text of the remaining articles and retained studies that met the inclusion/exclusion criteria. All data extraction and appraisals of retrieved studies were independently reviewed by MVD and BA, and all disagreements between the two reviewers were resolved through discussion and consensus. A third reviewer FAO adjudicated the differences that emerged in the selection of the final studies for inclusion. The summary of the selected studies was recorded, and this included: author, year of publication, number of children/number of mothers, age of children, factors associated with IYCF indicators, and quality assessment score.

### 2.4. Quality Assessment

The quality assessment of the review was based on the assessment tools of the National Heart, Lung, and Blood Institute of the National Institutes of Health (NIH) for quality assessment of Observational Cohort and Cross-Sectional Studies and Controlled Intervention Studies [26]. The checklist consists of 14 items that evaluate the external validity (based on potential selection bias) and internal validity (based on potential measurement biases and confounding) of observational studies. After the initial assessment of all reviewed studies, the items were further collapsed into eight quality-appraisal criteria: sample size, sampling methodology, responses rate, outcome measures, statistical analyses, study limitation, ethical consideration, and control for confounding. Scores assigned to each reviewed study range from zero to 14 points (zero if none of the criteria were met and 14 points if all criteria were met). The sum of points awarded represented the overall quality of the study. Studies were rated as good (≥11), medium (6–10), and poor (≤5). A low-quality rating implies a high risk of bias in the study and vice versa. Research has indicated that the NIH checklist is a comprehensive tool for assessing the risk of bias in observational and experimental studies [27,28,29].

## 3. Results

**Figure 1:**
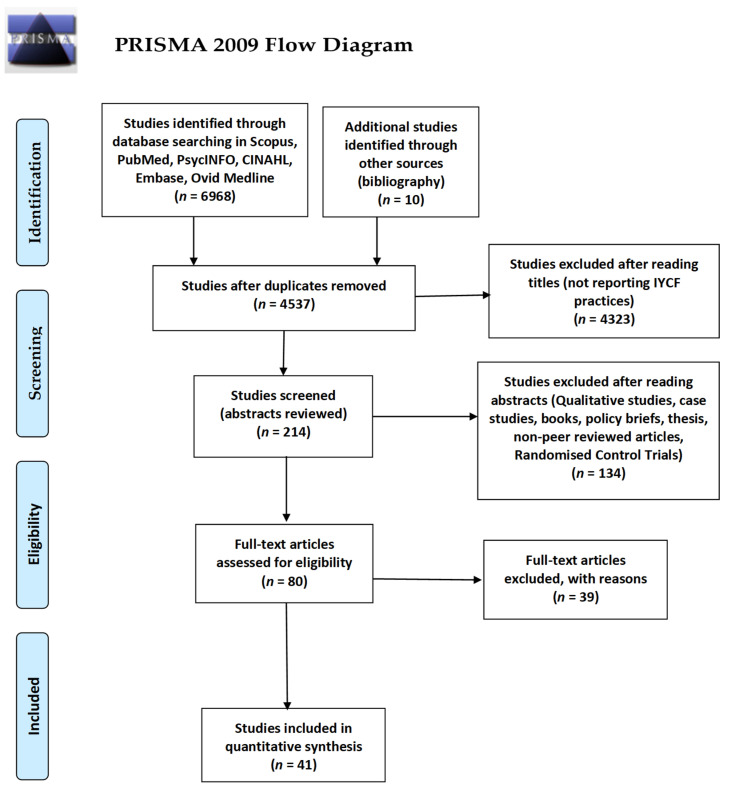
Flow chart for the study selection based on PRISMA 2015 guidelines.

A total of 6968 articles were retrieved from the six databases. A manual search of the bibliographic references of the retained articles identified 10 additional articles. After the removal of duplicates, 4537 articles were retained. The screening of the titles in the first screening phase resulted in the exclusion of 4323 articles. Further screening of the resulting 214 abstracts led to the exclusion of another 134 articles. In the final screening phase, the full texts of the remaining 80 articles were reviewed, and a further 39 articles were excluded. After the entire screening, 41 articles met the inclusion criteria and were thereby retained, as shown in Figure 1.

### 3.1. Characteristics of the Study

Table A1 and Table A2 demonstrates the summary of the studies included in this review. Of the studies conducted, nine studies were conducted at the national level, and 32 studies were conducted at the regional level. Sample sizes ranged from 77 mothers/children to 94,401 mothers/children. The criteria used to evaluate the quality of the included studies demonstrated that all the 41 studies were of medium quality. The details of the specific scores are provided in Supplementary Table S2. The extensive details of the studies have been provided in Supplementary Tables S3–S12. During the search, three randomized control trials (RCTs) were found. However, the RCTs were not included in the review, as their sampling procedures, study design, methodology, implementation, and the quality assessment criteria were different from observational studies.

### 3.2. Evidence from the Reviewed Studies

As shown in Table A1 and Table A2, the most consistent factors associated with the IYCF indicators included (i) socioeconomic factors such as family characteristics such as marital status, socioeconomic status/standard of living, family type/size and access to media sources like newspapers, radio, and television; (ii) child characteristics such as sex, birth status (pre-term, term, post-term), perceived size of the baby at birth, preceding birth interval, and birth order of the child; (iii) maternal characteristics such as maternal age, maternal age at marriage, education/literacy level, employment status, power over earnings, power over household purchases, type of caste or tribe, and religion and parity; (iv) community-level characteristics such as place of residence (urban or rural); and (v) health service factors such as breastfeeding counselling, registration for antenatal care (ANC), number of ANC visits, place of birthing, type of birthing assistance, and mode of birthing.

#### 3.2.1. Factors Associated with EIBF

The review showed that higher socioeconomic status [28,29], higher maternal education [28,30,31,32,33,34,35,36], maternal employment [24], access to media sources [24], term/post-term birth [32,33,34] [34,36,37], and maternal age (≥20 years) [32,36] were associated with EIBF. Similarly, a receipt of breastfeeding counselling [32,34,37], frequent ANC visits [31] (≥3 [36], ≥4 [30], ≥7 [23,24]), health facility birthing [30,35,37,38], births assisted by health professionals [28], and vaginal birthing [30,32,33,34,38] were associated with EIBF. However, rural/urban residence [35] and caesarean birthing [30] were associated with delayed initiation of breastfeeding.

#### 3.2.2. Factors Associated with EBF Less than Six Months of Age

In the review, we found that middle or higher socioeconomic status [25,39,40,41], nuclear family [42,43,44], small family size [42,45], male children [17,46], female children [47], preterm birth [43], smaller babies at birth [44], lower birth order [30], maternal age [23,48] (20–25 years [37,39]), higher maternal education [42,46,49,50,51,52,53], maternal unemployment [38,45], and multiparity [39] were associated with EBF. Similarly, low socioeconomic status [38], low maternal education [38,43], employed mothers [44], and primiparity [51] and breastfeeding counselling [49,54,55] were also associated with EBF. Additionally, some studies found that young maternal age (15–24 years [53], <20 years [44]), low socioeconomic status [53], and urban residence [44] were associated with low EBF. In contrast, breastfeeding counselling [54,56,57], registration for ANC [39,41], number of ANC visits (≥3 [27,48,51], ≥4 [51], ≥7 [48]), hospital birthing [30,41], access to the type of birthing assistance, and vaginal birthing [26,33,44] were associated with EBF.

In contrast, factors such as high socioeconomic status [44], male children [46], early marriage of parents [46], young maternal age (≤20 years) [50], low maternal education [25,46], primiparity [50], employed mothers [44,46], less frequent ANC visits (≤4) [46], caesarean birthing [46], delayed initiation of breastfeeding [46], a lack of knowledge about EBF [46], and poor maternal counselling regarding EBF [46] were barriers to EBF. Additionally, higher socioeconomic status [17,26,58], nuclear family [59], higher birth order [17], female children [17], larger baby size at birth [17], higher maternal education [17,25,52,58,60], consecutive birthing (≤24 months) [50], rural residence [17,44], health facility birthing [24], and birthing in summer [59] were negatively associated with EBF whereas one study Mahmood et al. [56] found no association between maternal factors and EBF.

#### 3.2.3. Factors Associated with Continued Breastfeeding at One Year (12–15 Months)

Only one study by Kumar et al. [40] considered continued breastfeeding at one year, and the study showed that maternal age (21–30 years) and joint family were associated with continued breastfeeding at one year.

#### 3.2.4. Factors Associated with the Introduction of Solid, Semi-Solid, or Soft Foods, 6–8 Months

In the reviewed studies, we found that middle or high socioeconomic status [18,57,58,61], access to media [24,61], male children [30], high birth order [18,58], high maternal education [30,31,37,57,58], high parity [57], urban residence [18], frequent ANC visits [37,60] (≥4 [18], ≥6 [61], >7 [24]), health professional advice [60], hospital birthing [57,58], and vaginal birthing [57] were associated with introduction of solid, semi-solid, or soft foods.

#### 3.2.5. Factors Associated with MDD, 6–23 Months

The review demonstrated that MDD was associated with higher socioeconomic status [18,22], media exposure [60], higher maternal education [18], woman’s autonomy over power of earnings [18], higher birth order [18,22], urban residence [22], and frequent ANC visits (≥4) [18]. Low socioeconomic status [61], low maternal education [61], lower media exposure [61], and fewer ANC visits (<6 to none) [61] were associated with inadequate MDD.

#### 3.2.6. Factors Associated with MMF, 6–23 Months

The review showed that MMF was associated with higher socioeconomic status [18], media exposure [60], male gender [22], higher birth order (≥2) [18], higher maternal education [18,22], woman’s autonomy over finances [18], urban residence [22], health professional-assisted births [18], and frequent ANC visits (≥4) [18]. Low socioeconomic status [61], less exposure to media [61], low maternal education [61], less power over household decision making [61], and less frequent ANC visits (<6 to none) [61] were associated with inadequate MMF.

#### 3.2.7. Factors Associated with MAD, 6–23 Months

The reviewed studies demonstrated that MAD was associated with richer households [18,22], male children [22], higher birth order [18,22], older maternal age (≥25 years) [18], urban residence [22], health facility birthing [18,58], and ANC visits (≥4) [18]. Lower socioeconomic status [61], low maternal education [61], less exposure to media [61], less power over household decision making [61], and less frequent ANC visits (<6 to none) [61] were associated with inadequate MAD.

#### 3.2.8. Factors Associated with Continued Breastfeeding at Two Years (20–23 Months)

In the reviewed studies, we found that high socioeconomic status [62], high maternal education [63], private hospital birthing [62], and increasing urbanicity [63] were associated with breastfeeding discontinuation before 24 months. Male children [62,64], higher birth order (≥2) [62], rural women [62,64], younger mothers (≤20 years) [64], and increasing maternal age at childbirth [62] were associated with continued breastfeeding at two years. Additionally, frequent ANC visits [62] and birthing assistance by a friend [64] were associated with continued breastfeeding at two years.

#### 3.2.9. Factors Associated with Predominant Breastfeeding Less than Six Months of Age

Srivastava et al. [25] demonstrated that factors associated with predominant breastfeeding included lower socioeconomic status, lower maternal and paternal education, fewer ANC visits (<3), and fewer TT vaccinations.

#### 3.2.10. Factors Associated with Bottle Feeding, 0–23 Months

Patel et al. [24] found that the factors associated with bottle feeding included birthing assisted by non-health professionals, smaller birth size, higher socioeconomic status, higher media exposure, maternal employment, higher maternal education, and urban residence.

## 4. Discussion

The review showed that the most common factors associated with appropriate IYCF indicators were middle/higher socioeconomic status, frequent exposure to media, child gender (male), and higher birth order (≥2). Other common factors included maternal age, higher maternal education, employment status (housewife and employed), multiparity and a higher number of ANC visits (≥3), health facility birthing, and vaginal birthing.

The association between higher socioeconomic status and IYCF practices reported in this review is consistent with studies conducted in Bangladesh [65,66] and Pakistan [67,68]. Higher socioeconomic status and better media exposure [69] may translate into better awareness about appropriate IYCF practices, which may in turn influence a mother’s decision to improve child-related health outcomes, including nutrition [70]. Our review also showed that male children and those with higher birth order (≥2) were more likely to be appropriately fed. Similarly, a study from Bangladesh [71] has found significant association between birth order and IYCF practices, while studies from Pakistan [72,73,74] and Nepal [75] have reported that higher birth order was associated with inappropriate IYCF practices, whereas a study from Sri Lanka [76] has found no significant association between birth order and IYCF practices. Higher birth order may reflect a more experienced mother in relation to appropriate infant feeding, as the mother may be more aware of what type of food to give to the child at every stage of growth and development [77]. In India, evidence suggests that there is an increasing desire for male children, and this male preference may have an impact on child health and development [78]. It is uncertain to what extent this cultural practice may be affecting infant and young child nutrition in the population, and future research may consider the impact of male preference on infant and young child nutrition in the country.

Previous studies from Pakistan, Bangladesh, and Nepal have reported that maternal characteristics such as higher maternal age (≥25 years) [67], higher maternal education [67,79], employment status [80,81,82], parity (≥2), and maternal autonomy in finance [83] were associated with appropriate IYCF practices. Similarly, our review showed that maternal characteristics (maternal age, maternal education, employment status, parity, and maternal autonomy) were associated with appropriate IYCF indicators. Globally, women empowerment indicators have been shown as a major determining factor for optimal child growth and development [80,81,84,85]. Higher maternal education increases women’s opportunities for employment, household earnings, and autonomy; empowers the woman to make informed child health-related decisions such as the uptake of appropriate IYCF information; and improves the woman’s attitude towards seeking appropriate child health support for appropriate IYCF [82]. While some studies have shown that women in employment have advantages of improving earnings/confidence and subsequent health-related decisions for IYCF [67,86,87,88], other studies have indicated that “stay-at-home” mothers (housewives) also have advantages for appropriate IYCF [89,90]. Being a housewife allows the mother to have enough time and support for careful consideration of appropriate IYCF practices, and the mother is not distracted by external work activities compared to the mother in employment [89,90].

Globally, numerous studies from LMICs have shown that health service factors (including breastfeeding counselling in health facilities, ANC visits, and health facility birthing and normal vaginal births) are strongly associated with appropriate IYCF [91,92,93,94]. Consistent with past studies, the review showed that health service factors were associated with appropriate IYCF in India. Empirical evidence suggests that increased access to health services for women can improve many maternal and child health domains. These areas include access to relevant information and support for behavior change for both mother and child; better opportunities for making informed decisions about preventive maternal and child health measures; and possibly providing an entry point for better household decision-making process about child health [95].

### 4.1. Policy Implications of the Study Findings

In India, information relating to IYCF policy initiation and implementation has been extensively documented in previously published studies [2,4,17,18,35]. Briefly, we highlight various national policies and programs that are being implemented in India to improve child nutrition. These policies are in support of the major IYCF programs introduced nationally to support the Integrated Child Development Scheme (ICDS) [96]. Efforts have been made to improve the coordination between the national and state level ICDS implementation through recommendations in the Twelfth five-year plans (2012–2017), such as organization “Village Health and Nutrition Days” to promote the uptake of appropriate IYCF practices [97]. However, the strategic decision to restructure the focus of ICDS to children under three years of age to target the increased uptake of key IYCF indicators to guide policies and laws still requires revisions to document any major impact on the nutritional status of children. Additional policies to support ICDS have been implemented, which include the Pradhan Mantri Surakshit Matritva Abhiyan (PMSMA) [98], Pradhan Mantri Matru Vandana Yojana (PMMVY) [99], LaQshya programme [100], HealthPhone [101], cash transfer schemes such as Janani Suraksha Yojna (JSY) [102], and recent amendments to the Maternity Benefit Act, 1961 (now known as the Maternity Benefit Amendment Act, 2017) [103]. The impacts of these initiatives across regional and national India are yet to be publicly documented, as current estimates of IYCF showed that these indicators are below expected levels in many regional areas [17,18]. Similarly, employed mothers still face significant resistance around workplace breastfeeding [104]. Streamlining India’s IYCF policies and programs may likely have maximal impacts due to the positive impacts of policy-driven and targeted approaches for IYCF interventions employed in other contexts. For example, Sri Lanka now has a strong focus on policy directives on mass communication for maternal education and special consideration for working mothers around IYCF. This initiative has been shown to significantly increase the uptake of IYCF practices across all levels of the population [105].

Moreover, the improvement of breastfeeding and complementary feeding participation of Indian women would also require an increase in female education as articulated in the Sustainable Development Goals [106] as well as the pragmatic implementation of the World Breastfeeding Trends Initiative (WBTi) recommendations [107]. One of the recommendations includes the establishment of standard BFHI centers for promoting, protecting, and supporting breastfeeding. While the Government of India has established programs, including Mother’s Absolute Affection (MAA) [108] and the Prime Minister’s Overarching Scheme for Holistic Nourishment (POSHAN) Abhiyaan [109] to promote, protect, and support breastfeeding, intensifying efforts to improve the uptake of these programs is still needed [108].

Worldwide, husband/partner and family (e.g., grandmother) support for breastfeeding is essential for appropriate IYCF practices in the household [110]. With the appropriate knowledge, these key family members provide an emotional, psychological, and physical support system for new mothers [111]. A recent systematic review indicated that appropriate partner breastfeeding support (in terms of verbal encouragement to new mothers from their partners, assistance in preventing and managing breastfeeding difficulties, and/or assistance with household/child care duties) influenced new mothers’ decision to initiate, continue, or cease breastfeeding in the early postnatal period [112]. The important role of grandmothers in influencing maternal IYCF decisions has also been documented in Malawi [113], Nigeria [114], and internationally [115]. In India, the new Home-Based Care for Young Child initiative introduced in 2018 aims to strengthen the nutritional levels of children through structured home visit counselling of the caregivers and mothers [116]. This initiative is focused on a community-based approach with family units as the center of care to improve the IYCF uptake of children. However, these policy interventions that seek to improve IYCF practices in India should also consider maximizing the aspects of a family support system to improve the IYCF uptake.

### 4.2. Strengths and Limitations

This systematic review is a comprehensive search of existing literature on the association between socioeconomic, demographic, and health service factors and IYCF practices in India to inform targeted policy interventions. The strengths of our systematic review lie in the exhaustive search through extensive databases utilizing broad search strings and having two independent reviewers undertake the study selection, reanalyzing the studies to be included in the review based on the inclusion and exclusion criteria through discussion and consensus, as well as quality assessment. However, the study has limitations. First, the review was limited to quantitative studies with the exclusion of qualitative studies to appropriately answer the research question. The inclusion of the qualitative studies would allow for triangulation of results and provide alternative explanations for the findings [117]. Future studies should be conducted to highlight the in-depth reasons for the varied patterns of IYCF feeding practices in India. Second, the exclusion of studies not written in English and those published in multiple centers across different countries (including India) would have limited our evidence, as those excluded studies may have had additional information. Third, there may have been a publication bias, given that grey literature was excluded. Fourth, most of the included studies were cross-sectional studies, and recall bias is potentially inherent in the findings due to the nature of the data collection. Finally, the evidence from this study may be limited, given the study design. Future experimental studies that investigate the association between the socioeconomic, demographic, and health service factors and IYCF practices in India may be needed.

## 5. Conclusions

In India, our review has shown that the factors associated with IYCF practices are multi-factorial. There is a need for a multi-sectorial strategy that hinges on both facility- and community-based approaches at the sub-national and national levels to improve IYCF practice in India. These public health measures should not only include IYCF education or counselling sessions for mothers, but should also involve other important hierarchical (socioeconomic, demographic, and health service) factors in the households and community to improve childhood feeding.

## Data Availability

No new data were created or analyzed in this study. Data sharing is not applicable to this article.

## Supplementary Materials

The following are available online at https://www.mdpi.com/2072-6643/13/3/825/s1, Table S1. PRISMA 2009 Checklist; Table S2. Quality Assessment Score; Table S3. Early initiation of breastfeeding; Table S4. Exclusive breastfeeding; Table S5. Continued breastfeeding at 1 year; Table S6. Continued breastfeeding at 2 years; Table S7. Predominant breastfeeding; Table S8. Bottle feeding; Table S9. Introduction to solid, semi-solid or soft foods; Table S10. Minimum Dietary Diversity; Table S11. Minimum Meal Frequency; Table S12. Minimum Acceptable Diet.

## Appendix A

**Table A1:** Summary of selected studies: Breastfeeding indicators.

Author; Year	Number of Children/Number of Mothers/Age of Children	Geographical Region	Study Design	Factors Associated with Breastfeeding Indicators						Study Strengths	Study Limitations	Quality Assessment Score
				EIBF/Timely Initiation of Breastfeeding	EBF	Continued Breastfeeding at One Year	Continued Breastfeeding at Two Years	Predominant Breastfeeding	Bottle Feeding			
Pariya et al.; 2020 [32]	*n* = 97 mothers; Age of children is not mentioned	Kolkata, West Bengal	Descriptive, observational, institution-based, cross-sectional study	High maternal education, higher maternal age at marriage (≥20 years), vaginal/vaginal assisted delivery, higher number (≥3) of antenatal (ANC) visits, advice regarding breastfeeding practice and term/post-term baby						Pre-designed, pre-tested semi-structured questionnaire was used.	The sample is not representative of the national population and is notably small. There could have been Berkesonian bias in the results. Causal relationship could not be established due to the nature of the study.	9
Bhanderi et al.; 2019 [46]	330 infants; Six months–one year	rural community of central Gujarat	Community-based cross-sectional study		Early marriage of parents, low maternal and paternal education, male child, Christian religion, employed mothers, a smaller number of ANC visits (≤4), operative delivery, late initiation of breastfeeding, not feeding colostrum, lack of knowledge about EBF, and poor counselling of mother regarding EBF were negatively associated.					Study was conducted in the community with adequate sample size and zero nonresponse, it has good external validity; thus, findings could be generalized to other populations of the state.	Causal relationship could not be established due to the nature of the study. There could also have been a possibility of recall bias. The study findings represent only a small region of India, and they do not represent the national population of India.	9
Sultania et al.; 2019 [38]	1000 women; Age of children is not mentioned	S.S. Hospital, Banaras Hindu University, Varanasi.	Cross-sectional, questionnaire-based study	Normal vaginal delivery and hospital delivery promoted EIBF.	Low maternal education, lower socio-economic status, and unemployed mothers.					A pre-designed, self-administered, standardized questionnaire was used	Causal relationship could not be established due to the cross-sectional nature of the study. The study findings are not representative of the national population of India, as they only represent a small community of India. There could also be a possibility of recall bias.	8
Senayake et al.; 2019 [35]	94,401 mothers; 0–23 months	India	Cross-sectional study	Higher maternal education, frequent ANC visits (≥4), and health facility delivery were positively associated. Urban mothers with health facility delivery were associated with high EIBF, whereas those with caesarean section were negatively associated. Similarly, mothers residing in the North-Eastern, Southern, Eastern, and Western regions were also associated with higher EIBF. Birthing through caesarean, receiving delivery assistance from non-health professionals, and rural area residence of the Central region were associated with delayed initiation of breastfeeding in all populations.						First, possible effect of selection bias is unlikely to impact the study findings based on the nationally representative nature of the sample size and the high response rates (94–99.6%). Second, the NFHS-4 data, including the study factors and EIBF were collected by trained personnel who used standardized questionnaires to ensure consistency across all Indian states and territories. Finally, our study provides relevant contextual evidence on key modifiable determinants of EIBF in one of the world’s largest populations.	A clear temporal association between the study factors and EIBF cannot be established due to the cross-sectional study. There could also have been recall bias in the study. There could also have been measurement bias leading to an overestimation or underestimation of factors. The information on the study factors and outcome variable were based on self-reporting, and this is a source of recall or measurement bias, which could result in an overestimation or underestimation of the association between the study factors and EIBF. Additionally, lack of assessment of unmeasured confounding factors could have also influenced the outcomes.	9
Randhawa et al.; 2019 [41]	370 mothers; Age of children is not mentioned	Badungar, a semi-urban area in Patiala city, Punjab	Community-based cross-sectional study		High maternal education, high socio-economic status, nuclear status of family, history of ANC registration, and health facility delivery were positively associated					A pre-designed, pre-tested semi-structured questionnaire was used.	Causal relationship could not be established due to the cross-sectional nature of the study. The study findings are not representative of the national population of India, as they only represent a small community of India. There could also be a possibility of recall bias due to the self-reporting and estimation based on the mothers’ recall.	7
Panigrahi et al.; 2019 [45]	160 mothers-infant pairs; 6–12 months	Slums of Bhubaneshwar, Odisha	Community-based cross-sectional study		Being a housewife, smaller family, ≥3 antenatal visits, and ≥3 postnatal visits were positively associated.					Response rate of 96.4% was high. The study thus had good external validity, and the findings can be generalized to the state population.	A causal relationship could not be established due to the cross-sectional nature of the study. There is a possibility of recall bias due to the nature of the reporting. There could have been an overestimation or under estimation of the outcome variables.	9
Matthew et al.; 2019 [53]	527 women-infant (<6 months) pairs	PSG Institute of Medical Sciences and Research, Coimbatore	Cross-sectional study		Younger maternal age (15–24 years), lower socio-economic status was negatively associated.					A large number of demographic and clinical data were collected, which can influence the association of duration of EBF. The same interviewer collected all information, which reduces the inter observer bias. Advanced statistical methods were employed to analyze the association of socio-demographic and clinical correlates with EBF.	A causal relationship could not be established due to the cross-sectional nature of the study. There is a possibility of recall bias due to the nature of the reporting. Additionally, the population is hospital-based and does not represent the national population.	8
Ogbo et al.; 2019 [17]	21,352 mother-infant pairs; 0–5 months	India	Cross sectional study		Higher birth order (North, Central, North-East) higher maternal education (South), other backward classes (West), female child (South), perceived to be large (South), rural mothers (West), higher socio-economic status (Central) were negatively associated. Higher maternal education (Central), scheduled tribe (East, North-East), caesarean delivery (North-East), currently married (North East), frequent ANC visits (≥4) (North) were positively associated.					Data collection had high response rates (from 94.0 to 99.6% across the states of India), reducing the potential effect of selection bias. Second, the India DHS data were collected by skilled personnel using standardized questionnaires, which ensured that the data collected were consistent across the states and territories of India. Lastly, the study provided evidence on important modifiable factors associated with EBF in the world’s second largest populations to help nutrition experts in the country advocate for effective policies and intervention services to improve EBF in India.	A temporal relation could not be established due to the cross-sectional nature of the study. There could be some recall bias due to the self-reporting. There could also be a measurement bias due to the over-reporting or under-reporting of the factors. All the confounding factors were not considered when conducting the study.	9
Chhetri et al.; 2018 [47]	137 working mothers; 0–6 months	Udupi taluk, Karnataka	Community-based cross-sectional study		High maternal and paternal education, place of delivery (private hospital), female child, frequency of breastfeeding per day, practice of expressing and storing breastmilk before leaving for work and breaks during working hours were found to be positively associated.					A validated, pre-designed questionnaire was used.	A temporal relation could not be established due to the cross-sectional nature of the study. Recall bias may have influenced the outcomes.	7
Nishimura et al.; 2018 [48]	1292 mothers; 0–12 months	Mysore, Karnataka	Cross-sectional study		Higher/increasing maternal age, lower maternal education, and frequent ANC visits (7–10) were positively associated.					The large sample size and low loss to follow up rate confers greater statistical power and generalizability. The questionnaire was based on validated items from the India’s National Family Health Survey-3	Recall bias could be there due to the nature of the interviews. There could be some confounding factors, which could influence the outcomes of the study. Additionally, the results are not generalizable to the national population.	10
Velusamy et al.; 2017 [59]	1088 mothers; 0–6 months	Vellore, South India	Community-based prospective birth cohort study (combining data from three studies)		High maternal education, pucca type of house, two or more number of children in the family, nuclear family structure and birth during summer were negatively associated.					Prospective design of the study was a major strength. Further, pooling data from three similar birth cohort studies resulted in larger sample size of 1088, reducing the risk of chance findings and adding statistical power to the analyses of relevant determinants. Rigorous follow-up allows information for most children to be available, hence reducing the bias due to attrition.	Study was not designed to assess the determinants of exclusive breastfeeding. Additional factors needed to be assessed for key factors associated with exclusive breastfeeding would likely include additional relevant factors that were not collected as a part of the existing studies. Additionally, missing information on antenatal visits, prelacteal feeding, time of initiation of breastfeeding, maternal nutrition, and vaccination schedule did not allow the investigators to investigate these factors.	10
Veeranki et al.; 2017 [23]	1294 mother-infant pairs; 0–12 months	Mysore, Karnataka	Prospective cohort study	Maternal dissatisfaction with the infant’s gender had higher odds of delayed initiation of breastfeeding. Mothers with frequent ANC visits (7–10) and assistance during breastfeeding were increasingly associated with timely initiation of breastfeeding.	Older maternal age was negatively associated with nonexclusive breastfeeding. High maternal education was positively associated with non-EBF.					Strong design of prospective cohort and large sample size, which allowed for examining sociodemographic and delivery characteristics associated with strong statistical power and analysis and minimal recall bias.	The findings are not generalizable to the national population of India. There could have been recall bias in the study. Additionally, there have been other confounding factors such as breastfeeding problems of mothers, previous reproductive history (e.g., number of abortions and neonatal deaths), feeding preference of family members, and feeding practices of friends, which were not considered for the study and could have influenced the outcomes.	8
Oakley et al.; 2017 [63]	7848 children; <6 year of age	Ranga Reddy district, southern India	Cross-sectional study		High maternal education, higher socioeconomic status was positively associated with early termination of EBF.		High maternal education, increasing urbanicity were positively associated with breastfeeding discontinuation before 24 months			Validated questionnaire was used. Advanced statistical methods were employed to run the analysis.	Temporal association between outcome and the study factors may not be established due to the nature of the study. There is a possibility of recall bias due to the nature of data collection. There could have been differential misclassification bias due to the nature of the factors.	8
Mehta et al.; 2017 [64]	7534 women	India	Cross-sectional				Male children, rural women, younger maternal age at marriage (17 years), delivery assistance by a friend were positively associated.			Response rate was high in the participants. Validated questionnaire was used for the analysis.	Recall bias may have influenced the results. The temporality could not be established due to cross-sectional study. There could have been social desirability bias due to women feeling the pressure of answering in a certain way. Additionally, there could be other factors like women’s occupation, which could be influencing the results.	8
Das et al.; 2016 [54]	20,793 mothers of 0–5-month-old children and 10,130 mothers of 6–8-month-old children	Bihar	Cross-sectional study		Winter nursing and breastfeeding counselling were positively associated					The large sample size allowed for robust analysis for multiple covariates simultaneously in the regression analyses and to perform age subgroup analyses. Moreover, as the LQAS surveys were conducted across multiple rounds during different times of the year, analyses of the seasonal trends without being concerned about the sample size were possible. Additionally, a uniform protocol and rigorous training methodology was implemented across the survey regions and rounds, which reduced the between-interviewer variations and improved the quality of collected data.	Temporal relation could not be established due to the cross-sectional nature of the study. The study findings are not generalizable to the national population. There could also have been social desirability bias, measurement bias, and the recall bias due to the nature of the study and the data collection.	8
Sharma et al.; 2016 [36]	210 infants; 0–12 months	tribal area of Madhya Pradesh	Community-based cross-sectional study	High maternal and paternal education and maternal employment status (housewife), higher socioeconomic status, counselling of mother during antenatal visits about need of breast feeding, hospital delivery, delivery conducted by trained person, and mother who received post-natal advice were positively associated						A pre-tested, validated, standard questionnaire was used.	The study findings are not representative of the national population of India. There could have been recall bias or misclassification bias. Additionally, the temporality cannot be established due to cross-sectional study.	8
Gupta et al.; 2015 [30]	194 mother-children pairs; 0–23 months	Delhi	Community-based cross-sectional study	Higher socio-economic status, government institution delivery, normal vaginal delivery was positively associated with EIBF. Caesarean delivery was associated with delayed initiation of breastfeeding.	Lower birth order, institutional delivery, normal vaginal delivery were positively associated.					A pre-validated standard questionnaire was used	The study findings are not representative of national Indian population. The temporality could not be established due to the cross-sectional nature of the study. There could have been recall bias and mis classification bias in the study.	7
Chandiok et al.; 2015 [44]	34,176 and 25,459 births in NFHS-1 and NFHS-3 respectively; 0–5 months	India	Community-based cross-sectional study		In the NFHS-1, infants perceived to be small size at birth and employed mothers were positively associated. Urban residence, younger maternal age (<20 years), high maternal education, higher SLI status, preceding birth interval (< two years), ANC care, were negatively associated. However, in the NFHS-3, rural residence, low maternal education, employed mothers, ANC care were negatively associated.					Use of validated questionnaire and nationally representative data set over two time points, very high survey response rates, low rates of missing and excluded data and appropriate adjustments for sampling design made in the analysis.	Causality cannot be established due to the cross-sectional nature of the study. There could be recall bias due to the data collection methods, and there could be misclassification error leading to under/overestimation of the results.	8
Choudhary et al.; 2015 [39]	1000 mothers; Age of children is not mentioned	postnatal care OPD in a tertiary care center- J.P.Haspital in Bhopal, Madhya Pradesh	Cross-sectional observational study		Maternal age (20–25 years), high maternal education, high socioeconomic status, multiparity, and availing ANC services were positively associated.					A predesigned, pretested questionnaire was used	The study findings cannot be generalized to the national Indian population. The causality cannot be established due to the cross-sectional nature of the study; recall bias and misclassification bias could also be there.	8
Gogoi et al.; 2015 [51]	136 children; 6–24 months	Dibrugarh, Assam	Cross-sectional study, Mixed method model		Mothers from nuclear family, primiparity, frequent ANC visits (≥4) were positively associated.					A predesigned, pretested questionnaire was used	The study findings could not be generalized to the national Indian population. The causality cannot be established due to the cross-sectional nature of the study; recall bias and misclassification bias could also be there.	7
Prasad et al.; 2015 [34]	350 children; 6–24 months	Pondicherry, India	Community-based cross-sectional study	Maternal age (21–25 years), high maternal education, employment status (housewife), vaginal delivery, full term delivery were positively associated.						A predesigned, pretested questionnaire was used	The study findings could not be generalized to the national Indian population. The causality cannot be established due to the cross-sectional nature of the study; recall bias and misclassification bias could also be there.	7
Srivastava et al.; 2014 [25]	1020 mothers; 0–6-week old infants	Two public hospitals, Lucknow, Uttar Pradesh	Prospective follow up cohort study		Low maternal and paternal education was negatively associated. Frequent ANC visits (≥3), mothers who had two vaccinations of tetanus toxoid (TT) during the antenatal period, Hindus and non-slum dwellers, medium socioeconomic status were positively associated			Low maternal and paternal education, fewer (<3) ANC visits, had fewer TT vaccinations, Muslims, slum dwellers, lower socioeconomic status were positively associated.		A predesigned, pretested questionnaire was used	The study was done in public hospitals among mothers from low socio-economic groups and therefore cannot be generalized for all institutional deliveries. The breastfeeding patterns can differ for home-delivered infants and institution delivered infants and hence cannot be generalized. The study could also not address the reason behind the low prevalence of exclusive breastfeeding in the study population.	9
Patel et al.; 2013 [33]	500 women who delivered live infants	Institutional Review Board of Indira Gandhi Government Medical College, Nagpur	Cross-sectional study	Higher maternal education, breastfeeding counselling, absence of obstetric problems, vaginal delivery, and high gestational age of newborn were positively associated						Pretested standardized questionnaire based on NFHS-3 was used to collect information on the mothers	The study findings could not be generalized to the national population of India. The causality cannot be established due to the cross-sectional nature of the study; recall bias and misclassification bias could also be there.	9
Mahmood et al.; 2012 [56]	123 woman-infant pairs; 0–12 months	Uttar Pradesh	Cross-sectional study		Multivariate logistic regression analysis showed that maternity and newborn care variables had no significant association.					Pretested, pre-validated questionnaire was used.	The temporality cannot be established due to the cross-sectional nature of the study; recall bias and misclassification bias could also be there due to the method of data collection. The study findings are not representative of the national population of India.	8
Kumar N. et al.; 2012 [40]	152 infants and mothers	Kasturba Medical College, Mangalore; in Coastal South India	Cross-sectional study		Middle to high socioeconomic status was positively associated.	Maternal age (21–30 years) and joint family mothers were positively associated.				Pretested, pre-validated questionnaire was used.	The temporality cannot be established due to the cross-sectional nature of the study; recall bias and misclassification bias could also be there due to the method of data collection. The study findings are not representative of the national population of India.	8
Bagul et al.; 2012 [49]	384 mother-children pairs	urban slum of Nagpur, Maharashtra	Community-based, cross-sectional study		High maternal education and breastfeeding counselling by health personals were positively associated.					Pretested, pre-validated questionnaire was used.	The temporality cannot be established due to the cross-sectional nature of the study; recall bias and misclassification bias could also be there due to the method of data collection. The study findings are not representative of the national population of India.	7
Radhakrishnan et al.; 2012 [42]	291 children; 6 months–2 years	Attyampatti Panchyat Union, Salem district, Tamil Nadu	Cross-sectional Study		Normal vaginal delivery, nuclear family, number of children (<2), smaller family size (<4) were positively associated					Pretested, pre-validated questionnaire was used.	The temporality cannot be established due to the cross-sectional nature of the study; recall bias and misclassification bias could also be there due to the method of data collection. The study findings are not representative of the national population of India.	8
Bhanderi et al.; 2011 [37]	300 children under five years of age	Petlad town, a semiurban area of Anand district, Gujarat, India	Community-based, cross-sectional study	High maternal education, ANC care, hospital delivery was positively associated with EIBF	Maternal age (22–26 years) was positively associated					Pretested, pre-validated questionnaire was used.	The temporality cannot be established due to the cross-sectional nature of the study; recall bias and misclassification bias could also be there due to the method of data collection. The study findings are not representative of the national population of India.	8
Patel et al.; 2010 [24]	20,108 children; 0–23 months	India	Cross-sectional study	The rate was higher for babies of employed mothers, frequent ANC visits (≥7), and mothers exposed to media such as radio and lower for babies delivered by caesarean section. The North-Eastern region continued to have the highest and the Central region to have the lowest rate.	High socioeconomic status and health facility delivery were negatively associated Normal vaginal or assisted delivery were positively associated. As compared with the Northern region, all other regions had higher rate.				Smaller babies and those born without the assistance of a health professional were negatively associated. Employed mothers, high maternal education, high socioeconomic status, urban residence, and those watching television had higher rate. As compared with the Northern region, the Central region had higher prevalence, whereas the North-Eastern and Western regions had lower rate.	Pretested, pre-validated questionnaire was used. A larger sample size was used, and it was nationally representative. The findings were generalizable to the Indian population.	The temporality cannot be established due to the cross-sectional nature of the study; recall bias and misclassification bias could also be there due to the method of data collection	9
Kunwar et al.; 2010 [52]	272 mothers; 6–8 months	Lucknow, Northern India	Cross-sectional hospital-based survey		High maternal education was positively associated					A pre-validated, pre-tested questionnaire was used.	The temporality cannot be established due to the cross-sectional nature of the study; recall bias and misclassification bias could also be there due to the method of data collection. The study findings are not generalizable to national population of India.	7
Jayant et al.; 2010 [31]	300 children; 0–5 years	Pravara Rural Hospital, Loni, Maharastra	Cross-sectional descriptive study	High maternal education was positively associated						A pre-validated, pre-tested questionnaire was used.	The temporality cannot be established due to the cross-sectional nature of the study; recall bias and misclassification bias could also be there due to the method of data collection. The study findings are not generalizable to national population of India.	7
Tiwari et al.; 2009 [43]	279 mother-infant pairs; 6–11 months	Gwalior, India	Community-based cross-sectional study		Preterm infants, normal birth weight infants, EIBF, ANC visits (≥3), high maternal education, and immunization visits were positively associated.					A pre-validated, pre-tested questionnaire was used.	The temporality cannot be established due to the cross-sectional nature of the study; recall bias and misclassification bias could also be there due to the method of data collection. The study findings are not generalizable to national population of India.	8
Kishore et al.; 2009 [55]	77 mother-infant pairs; 0–6 months	Haryana	Community-based cross-sectional study		Breastfeeding counselling was positively associated					A pre-validated, pre-tested questionnaire was used.	The temporality cannot be established due to the cross-sectional nature of the study; recall bias and misclassification bias could also be there due to the method of data collection. The study findings are not generalizable to national population of India.	9
Chudasama et al.; 2009 [50]	498 infants; 0–12 months	South Gujarat	Cross-sectional study		Factors associated with non-EBF/early weaning were primiparity, consecutive delivery interval (<24 months), maternal age (<20 years), and paternal occupation as labor					A pre-validated, pre-tested questionnaire was used.	The temporality cannot be established due to the cross-sectional nature of the study; recall bias and misclassification bias could also be there due to the method of data collection. The study findings are not generalizable to national population of India.	7
Malhotra et al.; 2008 [62]	31,645 children; 0–24 months	India	Cross-sectional study				Muslims, Sikhs and Christians, OBCs, increasing maternal education, higher SLI, private hospital deliveries were positively associated. Male child, rural residence, increasing maternal age at childbirth, higher birth order, ANC care were negatively associated.			A pre-validated, pre-tested questionnaire was used. Sample size was nationally representative.	The temporality cannot be established due to the cross-sectional nature of the study; recall bias and misclassification bias could also be there due to the method of data collection. The study findings are not generalizable to national population of India.	9

EIBF: Early initiation of Breastfeeding; EBF: Exclusive Breastfeeding; ANC: Ante Natal Clinic; NFHS 1–4: National Family Health Survey 1–4; SLI: Standard of Living Index; OPD: Out Patient Department; OBC: Other Backward Class.

**Table A2:** Summary of selected studies: Complementary feeding indicators.

Author; Year	Number of Children/Number of Mothers/Age of Children	Geographical Region	Study Design	Factors Associated with Complementary Feeding Indicators				Study Strengths	Study Limitations	Quality Assessment Score
				Introduction of Solid, Semi-Solid or Soft Foods	MDD	MMF	MAD			
Dhami et al.; 2019 [18]	69,464; 6–23 months children	India	Cross-sectional study	Higher socio-economic status (North, East), urban women (West), higher birth order (Central), frequent ANC visits (≥4) (Eastern and Central) were positively associated.	Higher socio-economic status (North, West, Central, North East), higher maternal education (North, Central), woman’s autonomy over power of earnings (South), higher birth order (North, South, East, West, North East), frequent ANC visits (≥4) (East) were positively associated.	Higher socio-economic status (South, East), higher maternal education (North, South, Central), woman’s autonomy over finances (Central), higher birth order (North, East, Central, North East), TBA- and health professional-assisted births (East), frequent ANC visits (≥4) (North, South, East, Central) were positively associated.	Higher socio-economic status (North, South), maternal age (≥25 years) (South), higher birth order (North, East, Central, North-East), health facility delivery (South), frequent ANC visits (≥4) (East, Central) were positively associated.	The study used the most recent and nationally representative data (NFHS-4) for India. The NFHS-4 data were obtained from a larger sample compared to previous national surveys, indicating that findings are more generalizable to the Indian population. The data used are comparable across regions in India, given that they were collected by trained personnel who used standardized questionnaires and methodology. The study findings are unlikely to be affected by selection bias as the survey yielded high responses rates, over 94%.	A temporal relation could not be established due to the cross-sectional nature of the study. There could be some recall bias due to the self-reporting. There could also be a measurement bias due to the over-reporting or under-reporting of the factors. All the confounding factors were not considered when conducting the study.	9
Ahmad et al.; 2017 [22]	326 children; 6–23 months	Jawaharlal Nehru Medical College, Aligarh Muslim University, Aligarh.	Community-based, cross-sectional study		Urban residence, high birth order and high standard of living index, (SLI) were positively associated	Urban residence, male child, higher maternal education were positively associated.	Urban residence, male child, higher birth order, higher SLI were positively associated.	A pre-validated standard questionnaire was used.	The study findings represent only a small section of the community and are not generalizable. There could have been recall bias. Additionally, the causality cannot be established due to the cross-sectional nature of the study.	8
Kakati et al.; 2016 [57]	250 infants 7–12 months	Kamrup district, Assam, India	Community based cross-sectional study	The infants born at Government. Institution, high socio-economic status, high maternal education, normal delivery, higher parity were positively associated.				A pre-validated standard questionnaire was used	The study findings are not generalizable to the national population of India. The causality could not be established due to the cross-sectional nature of the study. There could have been recall bias and information bias in the study.	8
Gupta et al.; 2015 [30]	194 mother-children pairs; 0–23 months	Delhi	Community based cross-sectional study	Higher maternal education and male child were positively associated.				A pre-validated standard questionnaire was used	The study findings are not representative of national Indian population. The temporality could not be established due to the cross-sectional nature of the study. There could have been recall bias and mis classification bias in the study.	7
Malhotra et al.; 2013 [60]	9241 children aged 6–18 months	India	Cross-sectional community-based study	ANC visits, health professional advice were positively associated	Media exposure to radio, reading newspaper were positively associated	Media exposure to radio, reading newspaper were positively associated		Pretested, pre-validated questionnaire was used.	The temporality cannot be established due to the cross-sectional nature of the study; recall bias and misclassification bias could also be there.	8
Patel et al.; 2012 [61]	15,028 last-born children; 6–23 months	India	Cross-sectional study	High socio-economic status, ≥6 ANC visits, mothers reading newspaper were positively associated. South, North East residents were positively associated.	Low socioeconomic status, low maternal education, lower exposure to media (radio, television or newspaper), fewer (<6 to none) ANC visits were negatively associated. East was negatively associated.	Women with child of 6–17 months, low education, did not read newspaper, less power in household decision making, less frequent ANC visits (<6 to none), lower socio-economic status were negatively associated. West and North were negatively associated.	Women with child of 6–17 months, low education, did not read newspaper, less power in household decision making, less frequent ANC visits (<6 to none), lower socio-economic status were negatively associated. East was negatively associated.	The ability to determine the most susceptible age group and the modifiable factors that affect inappropriate practices in a large sample size, which allows for control of confounders. The sample is nationally representative. A pre-validated questionnaire was used.	The temporality cannot be established due to the cross-sectional nature of the study; recall bias and misclassification bias could also be there due to the method of data collection.	9
Bhanderi et al.; 2011 [37]	300 children under five years of age	Petlad town, a semiurban area of Anand district, Gujarat, India	Community-based, cross-sectional study	High maternal education, ANC care were positively associated				Pretested, pre-validated questionnaire was used.	The temporality cannot be established due to the cross-sectional nature of the study; recall bias and misclassification bias could also be there due to the method of data collection. The study findings are not representative of the national population of India.	8
Rao et al.; 2011 [58]	200 mothers of children; aged 6–24 months	Dr TMA Pai Hospital Udupi and Dr TMA Pai Hospital Karkala and a public hospital, Regional Advanced Paediatric Care Centre, Mangalore.	Hospital-based cross-sectional study	Middle socioeconomic status, higher birth order, hospital delivery, higher maternal education were positively associated.			Hospital delivery was positively associated	Pretested, pre-validated questionnaire was used.	The temporality cannot be established due to the cross-sectional nature of the study; recall bias and misclassification bias could also be there due to the method of data collection. The study findings are not representative of the national population of India. Feed consistency was not taken into consideration for complementary feeding practices. Some of the questions asked were not open-ended. Additionally, the timescale over which the study was conducted was also a limitation.	7
Patel et al.; 2010 [24]	20,108 children; 0–23 months	India	Cross-sectional study	The rate was higher for women with frequent ANC visits (≥7) and for those who watched television. The rate was higher in the Southern, North-Eastern, and Eastern regions than in the Northern region.				Pretested, pre-validated questionnaire was used. A larger sample size was used, and it was nationally representative. The findings were generalizable to the Indian population.	The temporality cannot be established due to the cross-sectional nature of the study; recall bias and misclassification bias could also be there due to the method of data collection	9
Jayant et al.; 2010 [31]	300; 0–5 years	Pravara Rural Hospital, Loni, Maharastra	Cross-sectional descriptive study	High maternal education was positively associated				A pre-validated, pre-tested questionnaire was used.	The temporality cannot be established due to the cross-sectional nature of the study; recall bias and misclassification bias could also be there due to the method of data collection. The study findings are not generalizable to national population of India.	7

MDD: Minimum Dietary Diversity; MMF: Minimum Meal Frequency; MAD: Minimum Acceptable Diet; ANC: Ante Natal Clinic; NFHS 1 to 4: National Family Health Survey 1 to 4; SLI: Standard of Living Index.
