# Supplementary material for: Understanding the Enablers and Barriers to Appropriate Infants and Young Child Feeding Practices in India: A Systematic Review
Source: Nutrients. 2021 Mar 2;13(3):825. doi: 10.3390/nu13030825 (PMC7998710; doi:10.3390/nu13030825)
Supplement: Supplementary file 1 [file nutrients-13-00825-s001.pdf]

Supplementary Table S1. PRISMA 2009 Checklist

Section/topic	#	Checklist item	Reported on page #
**TITLE**			
Title	1	Identify the report as a systematic review, meta-analysis, or both.	1
**ABSTRACT**			
Structured summary	2	Provide a structured summary including, as applicable: background; objectives; data sources; study eligibility criteria, participants, and interventions; study appraisal and synthesis methods; results; limitations; conclusions and implications of key findings; systematic review registration number.	3
**INTRODUCTION**			
Rationale	3	Describe the rationale for the review in the context of what is already known.	4–5
Objectives	4	Provide an explicit statement of questions being addressed with reference to participants, interventions, comparisons, outcomes, and study design (PICOS).	4–5
**METHODS**			
Protocol and registration	5	Indicate if a review protocol exists, if and where it can be accessed (e.g., Web address), and, if available, provide registration information including registration number.	6
Eligibility criteria	6	Specify study characteristics (e.g., PICOS, length of follow-up) and report characteristics (e.g., years considered, language, publication status) used as criteria for eligibility, giving rationale.	6–8
Information sources	7	Describe all information sources (e.g., databases with dates of coverage, contact with study authors to identify additional studies) in the search and date last searched.	6–8
Search	8	Present full electronic search strategy for at least one database, including any limits used, such that it could be repeated.	6–8
Study selection	9	State the process for selecting studies (i.e., screening, eligibility, included in systematic review, and, if applicable, included in the meta-analysis).	6–8
Data collection process	10	Describe method of data extraction from reports (e.g., piloted forms, independently, in duplicate) and any processes for obtaining and confirming data from investigators.	8–9
Data items	11	List and define all variables for which data were sought (e.g., PICOS, funding sources) and any assumptions and simplifications made.	8–9
Risk of bias in individual studies	12	Describe methods used for assessing risk of bias of individual studies (including specification of whether this was done at the study or outcome level), and how this information is to be used in any data synthesis.	8–9
Summary measures	13	State the principal summary measures (e.g., risk ratio, difference in means).	8–9
Synthesis of results	14	Describe the methods of handling data and combining results of studies, if done, including measures of consistency (e.g., I^2 for each meta-analysis)	8–9
Risk of bias across studies	15	Specify any assessment of risk of bias that may affect the cumulative evidence (e.g., publication bias, selective reporting within studies).	8–9
Additional analyses	16	Describe methods of additional analyses (e.g., sensitivity or subgroup analyses, meta-regression), if done, indicating which were pre-specified.	8–9
**RESULTS**			

Study selection	17	Give numbers of studies screened, assessed for eligibility, and included in the review, with reasons for exclusions at each stage, ideally with a flow diagram.	10
Study characteristics	18	For each study, present characteristics for which data were extracted (e.g., study size, PICOS, follow-up period) and provide the citations.	10
Risk of bias within studies	19	Present data on risk of bias of each study and, if available, any outcome level assessment (see item 12).	10–13
Results of individual studies	20	For all outcomes considered (benefits or harms), present, for each study: (a) simple summary data for each intervention group (b) effect estimates and confidence intervals, ideally with a forest plot.	10–13
Synthesis of results	21	Present results of each meta-analysis done, including confidence intervals and measures of consistency.	10–13
Risk of bias across studies	22	Present results of any assessment of risk of bias across studies (see Item 15).	10–13
Additional analysis	23	Give results of additional analyses, if done (e.g., sensitivity or subgroup analyses, meta-regression [see Item 16]).	10–13
**DISCUSSION**			
Summary of evidence	24	Summarize the main findings including the strength of evidence for each main outcome; consider their relevance to key groups (e.g., healthcare providers, users, and policy makers).	14–16
Limitations	25	Discuss limitations at study and outcome level (e.g., risk of bias), and at review-level (e.g., incomplete retrieval of identified research, reporting bias).	17
Conclusions	26	Provide a general interpretation of the results in the context of other evidence, and implications for future research.	17
**FUNDING**			
Funding	27	Describe sources of funding for the systematic review and other support (e.g., supply of data); role of funders for the systematic review.	19

From: Moher D, Liberati A, Tetzlaff J, Altman DG, The PRISMA Group (2009). Preferred Reporting Items for Systematic Reviews and Meta-Analyses: The PRISMA Statement. PLoS Med 6(7): e1000097. doi:10.1371/journal.pmed1000097. For more information, visit: www.prisma-statement.org.

Supplementary Table S2. Quality Assessment Score

Author, Year	Was the research question or objective in this paper clearly stated?	Was the study population clearly specified and defined?	Was the participation rate of eligible persons at least 50%?	Were all the subjects selected or recruited from the same or similar populations (including the same time period)? Were inclusion and exclusion criteria for being in the study prespecified and applied uniformly to all participants?	Was a sample size justification, power description, or variance and effect estimates provided?	For the analyses in this paper, were the exposure(s) of interest measured prior to the outcome(s) being measured?	Were the exposure measures (independent variables) clearly defined, valid, reliable, and implemented consistently across all study participants?	Was the timeframe sufficient so that one could reasonably expect to see an association between exposure and outcome if it existed?	For exposures that can vary in amount or level, did the study examine different levels of the exposure as related to the outcome (e.g., categories of exposure, or exposure measured as continuous variable)?	Were the exposure measures (dependent variables) clearly defined, valid, reliable, and implemented consistently across all study participants?	Was the exposure(s) assessed more than once over time?	Were the outcome assessors blinded to the exposure status of participants?	Was loss to follow-up after baseline 20% or less?	Were key potential confounding variables measured and adjusted statistically for their impact on the relationship between exposure(s) and outcome(s)?	Quality Assessment Score
Pariya et al.; 2020	1	1	1	1	1	1	1	N/A	N/A	1	0	N/A	0	1	9
Bhanderi et al.; 2019	1	1	1	1	1	1	1	N/A	N/A	1	0	N/A	0	1	9
Sultania et al.; 2019	1	1	1	1	1	1	1	N/A	N/A	1	0	N/A	0	0	8
Senayake et al.; 2019	1	1	1	1	1	1	1	N/A	N/A	1	0	N/A	0	1	9
Randhawa et al.; 2019	1	1	1	1	0	1	1	N/A	N/A	1	0	N/A	0	0	7
Panigrahi et al.; 2019	1	1	1	1	1	1	1	N/A	N/A	1	0	N/A	0	1	9
Matthew et al.; 2019	1	1	1	1	0	1	1	N/A	N/A	1	0	N/A	0	1	8
Dhami et al.; 2019	1	1	1	1	1	1	1	N/A	N/A	1	0	N/A	0	1	9
Ogbo et al.; 2019	1	1	1	1	1	1	1	N/A	N/A	1	0	N/A	0	1	9
Chhetri et al.; 2018	1	1	1	1	0	1	1	N/A	N/A	1	0	N/A	0	0	7
Nishimura et al.; 2018	1	1	1	1	0	1	1	N/A	N/A	1	1	N/A	1	1	10
Velusamy et al.; 2017	1	1	1	1	0	1	1	N/A	N/A	1	1	N/A	1	1	10
Veeranki et al.; 2017	1	1	1	1	0	1	1	N/A	N/A	1	0	N/A	0	1	8
Oakley et al.; 2017	1	1	1	1	0	1	1	N/A	N/A	1	0	N/A	0	1	8
Mehta et al.; 2017	1	1	1	1	0	1	1	N/A	N/A	1	0	N/A	0	1	8
Ahmad et al.; 2017	1	1	1	1	0	1	1	N/A	N/A	1	0	N/A	0	1	8
Das et al.; 2016	1	1	1	1	0	1	1	N/A	N/A	1	0	N/A	0	1	8
Sharma et al.; 2016	1	1	1	1	1	1	1	N/A	N/A	1	0	N/A	0	0	8
Kakati et al.; 2016	1	1	1	1	1	1	1	N/A	N/A	1	0	N/A	0	0	8
Gupta et al.; 2015	1	1	1	1	0	1	1	N/A	N/A	1	0	N/A	0	0	7
Chandiok et al.; 2015	1	1	1	1	0	1	1	N/A	N/A	1	0	N/A	0	1	8
Choudhary et al.; 2015	1	1	1	1	1	1	1	N/A	N/A	1	0	N/A	0	0	8
Gogoi et al.; 2015	1	1	1	1	0	1	1	N/A	N/A	1	0	N/A	0	0	7
Prasad et al.; 2015	1	1	1	1	0	1	1	N/A	N/A	1	0	N/A	0	0	7
Srivastava et al.; 2014	1	1	1	1	0	1	1	N/A	N/A	1	1	N/A	1	0	9
Patel et al.; 2013	1	1	1	1	1	1	1	N/A	N/A	1	0	N/A	0	1	9
Malhotra et al.; 2013	1	1	1	1	0	1	1	N/A	N/A	1	0	N/A	0	1	8
Patel et al.; 2012	1	1	1	1	1	1	1	N/A	N/A	1	0	N/A	0	1	9
Mahmood et al.; 2012	1	1	1	1	0	1	1	N/A	N/A	1	0	N/A	0	1	8
Kumar N. et al.; 2012	1	1	1	1	1	1	1	N/A	N/A	1	0	N/A	0	0	8
Bagul et al.; 2012	1	1	1	1	0	1	1	N/A	N/A	1	0	N/A	0	0	7
Radhakrishnan et al.; 2012	1	1	1	1	0	1	1	N/A	N/A	1	0	N/A	0	1	8
Bhanderi et al.; 2011	1	1	1	1	1	1	1	N/A	N/A	1	0	N/A	0	0	8
Rao et al.; 2011	1	1	1	1	0	1	1	N/A	N/A	1	0	N/A	0	0	7
Patel et al.; 2010	1	1	1	1	1	1	1	N/A	N/A	1	0	N/A	0	1	9
Kunwar et al.; 2010	1	1	1	1	0	1	1	N/A	N/A	1	0	N/A	0	0	7
Jayant et al.; 2010	1	1	1	1	0	1	1	N/A	N/A	1	0	N/A	0	0	7
Tiwari et al.; 2009	1	1	1	1	1	1	1	N/A	N/A	1	0	N/A	0	0	8
Kishore et al.; 2009	1	1	1	1	1	1	1	N/A	N/A	1	0	N/A	0	1	9
Chudasama et al.; 2009	1	1	1	1	0	1	1	N/A	N/A	1	0	N/A	0	0	7
Malhotra et al.; 2008	1	1	1	1	1	1	1	N/A	N/A	1	0	N/A	0	1	9

Supplementary Table S3. Early initiation of breastfeeding

Author; Year	Number of children/number of mothers/ Age of children	Geographical region	Study design	Factors associated with EIBF/timely initiation of breastfeeding	Study Strengths	Study limitations	Quality assessment score
Pariya et al.; 2020 [1]	*n* = 97 mothers; Age of children is not mentioned	Kolkata, West Bengal	Descriptive, observational, institution based, cross-sectional study	High maternal education, higher maternal age at marriage (≥20 years), vaginal/vaginal assisted delivery, higher number (≥ 3) of antenatal (ANC) visits, advise regarding breastfeeding practice and term/post-term baby	A pre-designed, pre-tested semi-structured questionnaire.	The sample is not representative of the national population and is quite less. There could have been Berkesonian bias in the results. Causal relationship could not be established due to the nature of the study.	9
Sultania et al.; 2019 [3]	1000 women; Age of children is not mentioned	S.S. Hospital, Banaras Hindu University, Varanasi.	Cross-sectional, questionnaire-based study	Normal vaginal delivery and hospital delivery promoted EIBF.	A pre-designed, self-administered, standardized questionnaire was used	Causal relationship could not be established due to the cross-sectional nature of the study. The study findings are not representative of the national population of India as they only represent a small community of India. There could also be a possibility of recall bias.	8
Senayake et al.; 2019 [4]	94,401 mothers; 0–23 months	India	Cross-sectional study	higher maternal education, frequent ANC visits (≥ 4) and health facility delivery, were positively associated.<br><br>Urban mothers with health facility delivery was associated with high EIBF whereas those with caesarian section were negatively associated.<br><br>Similarly, mothers residing in the North-Eastern, Southern, Eastern and Western regions were also associated with higher EIBF. Birthing through caesarean, receiving delivery assistance from non-health professionals and rural area residence of the Central region were associated with delayed initiation of breastfeeding in all populations.	First, possible effect of selection bias is unlikely to impact the study findings based on the nationally representative nature of the sample size and the high response rates (94–99.6%). Second, the NFHS-4 data, including the study factors and EIBF were collected by trained personnel who used standardised questionnaires to ensure consistency across all Indian states and territories. Finally, our study provides relevant contextual evidence on key modifiable determinants of EIBF in one of the world's largest populations.	a clear temporal association between the study factors and EIBF cannot be established due to the cross-sectional study. There could also have been recall bias in the study. There could also have been measurement bias leading to an overestimation or underestimation of factors. The information on the study factors and outcome variable were based on self-reporting and this is a source of recall or measurement bias, which could result in an overestimation or underestimation of the association between the study factors and EIBF. Additionally, lack of assessment of unmeasured confounding factors could have also influenced the outcomes.	9
Veeranki et al.; 2017 [13]	1294 mother-infant pairs; 0–12 months	Mysore, Karnataka	prospective cohort study	Maternal dissatisfaction with the infant's gender had higher odds of delayed initiation of breastfeeding.<br><br>Mothers with frequent ANC visits (7–10) and assistance during breastfeeding was increasingly associated with timely initiation of breastfeeding	Strong design of prospective cohort and large sample size, which allowed for examining sociodemographic and delivery characteristics associated with strong statistical power and analysis and minimal recall bias.	The findings are not generalisable to the national population of India. There could have been recall bias in the study. Also, there have been other confounding factors such as breastfeeding problems of mothers, previous reproductive history (eg, number of abortions and neonatal deaths), feeding preference of family members, and feeding practices of friends which were not considered for the study and could have influenced the outcomes.	8
Sharma et al.; 2016 [18]	210 infants; 0–12 months	tribal area of Madhya Pradesh	community-based cross-sectional study	High maternal and paternal education and maternal employment status (housewife), higher socioeconomic status, counselling of mother during antenatal visits about need of breast feeding, hospital delivery, delivery conducted by trained person and mother who received Post-natal advice were positively associated	A pre-tested, validated, standard questionnaire was used.	The study findings are not representative of the national population of India. There could have been recall bias, misclassification bias. Also, the temporality can not be established due to cross-sectional study.	8
Gupta et al.; 2015 [20]	194 mother- children pairs; 0–23 months	Delhi	Community based cross-sectional study	Higher socio-economic status, government institution delivery, normal vaginal delivery was positively associated with EIBF.<br><br>Caesarian delivery was associated with delayed initiation of breastfeeding.	A pre-validated standard questionnaire was used	The study findings are not representative of national Indian population. The temporality could not be established due to the cross-sectional nature of the study. There could have been recall bias and mis classification bias in the study.	7
Prasad et al.; 2015 [24]	350 children; 6–24 months	Pondicherry, India	community-based cross-sectional study	Maternal age (21–25 years), high maternal education, employment status (housewife), vaginal delivery, full term delivery was positively associated.	A predesigned, pretested questionnaire was used	The study findings could not be generalised to the national Indian population. The causality cannot be established due to the cross-sectional nature of the study, recall bias and misclassification bias could also be there.	A predesigned, pretested questionnaire was used

Patel et al.; 2013 [26]	500 women who delivered live infants	Institutional Review Board of Indira Gandhi Government Medical College, Nagpur	cross-sectional study	higher maternal education, breastfeeding counselling, absence of obstetric problems, vaginal delivery, and high gestational age of newborn were positively associated	pretested standardized questionnaire based on NFHS-III was used to collect information on the mothers	The study findings could not be generalised to the national population of India. The causality cannot be established due to the cross-sectional nature of the study, recall bias and misclassification bias could also be there.	9
Bhanderi et al.; 2011 [33]	300 children under 5 years of age	Petlad town, a semiurban area of Anand district, Gujarat, India	community based, cross-sectional study	High maternal education, ANC care, hospital delivery was positively associated with EIBF	Pretested, pre-validated questionnaire was used.	The temporality cannot be established due to the cross-sectional nature of the study, recall bias and misclassification bias could also be there due to the method of data collection. The study findings are not representative of the national population of India.	8
Patel et al.; 2010 [35]	20,108 children; 0–23 months	India	cross-sectional study	The prevalence was higher for babies of employed mothers, frequent ANC visits (≥7) and mothers exposed to media such as radio and lower for babies delivered by caesarean section.<br><br>The North-Eastern region continued to have the highest and the Central region to have the lowest prevalence.	Pretested, pre-validated questionnaire was used. A larger sample size was used and it was nationally representative. The findings were generalisable to the Indian population.	The temporality cannot be established due to the cross-sectional nature of the study, recall bias and misclassification bias could also be there due to the method of data collection	9
Jayant et al.; 2010 [37]	300 children; 0–5 years	Pravara Rural Hospital, Loni, Maharastra	cross-sectional descriptive study	High maternal education was positively associated	A pre-validated, pre-tested questionnaire was used.	The temporality cannot be established due to the cross-sectional nature of the study, recall bias and misclassification bias could also be there due to the method of data collection. The study findings are not generalisable to national population of India.	7

Supplementary Table S4. Exclusive breastfeeding

Author; Year	Number of children/number of mothers/Age of children	Geographical region	Study design	Factors associated with Exclusive breastfeeding	Study Strengths	Study limitations	Quality assessment score
Bhanderi et al.; 2019 [2]	330 infants; 6 months–1 year	rural community of central Gujarat	community-based cross-sectional study	Early marriage of parents, low maternal and paternal education, male child, Christian religion, employed mothers, less number of ANC visits (≤4), operative delivery, late initiation of breastfeeding, not feeding colostrum, lack of knowledge about EBF, and poor counselling of mother regarding EBF were negatively associated.	study was conducted in the community with adequate sample size and zero nonresponse, it has good external validity thus, findings could be generalized to other populations of the state.	Causal relationship could not be established due to the nature of the study. There could also have been a possibility of recall bias. The study findings represent only a small region of India and they do not represent the national population of India.	9
Sultania et al.; 2019 [3]	1000 women; Age of children is not mentioned	S.S. Hospital, Banaras Hindu University, Varanasi.	Cross-sectional, questionnaire-based study	Low maternal education, lower socio-economic status and unemployed mothers.	A pre-designed, self-administered, standardized questionnaire was used	Causal relationship could not be established due to the cross-sectional nature of the study. The study findings are not representative of the national population of India as they only represent a small community of India. There could also be a possibility of recall bias.	8
Randhawa et al.; 2019 [5]	370 mothers; Age of children is not mentioned	Badungar, a semi-urban area in Patiala city, Punjab	Community -based cross-sectional study	high maternal education, high socio-economic status, nuclear status of family, history of ANC registration, and health facility delivery were positively associated	A pre-designed, pre-tested semi-structured questionnaire was used.	Causal relationship could not be established due to the cross-sectional nature of the study. The study findings are not representative of the national population of India as they only represent a small community of India. There could also be a possibility of recall bias due to the self-reporting and estimation based on the mothers' recall.	7
Panigrahi et al.; 2019 [6]	160 mothers-infant pair; 6–12 months	Slums of Bhubaneshwar, Odisha	community based cross sectional study	being housewife, smaller family, ≥3 antenatal visits, and ≥3 postnatal visits were positively associated.	response rate of 96.4% was high. The study thus had good external validity and the findings can be generalised to the state population.	a causal relationship could not be established due to the cross-sectional nature of the study. there is a possibility of recall bias due to the nature of the reporting. There could have been an overestimation or under estimation of the outcome variables.	9
Matthew et al.; 2019 [7]	527 women-infant (<6 months) pairs	PSG Institute of Medical Sciences and Research, Coimbatore	Cross-sectional study	younger maternal age (15–24 years), lower socio-economic status was negatively associated.	a large number of demographic and clinical data was collected which can influence the association of duration of EBF. The same interviewer collected all information which reduces the inter observer bias. Advanced statistical methods were employed to analyse the association of socio-demographic and clinical correlates with EBF.	a causal relationship could not be established due to the cross-sectional nature of the study. there is a possibility of recall bias due to the nature of the reporting. Also, the population is hospital based and does not represent the national population.	8
Ogbo et al.; 2019 [9]	21,352 mother infant pairs; 0–5 months	India	Cross sectional study	Higher birth order (North, Central, North-East) higher maternal education (South), other backward classes (West), female child (South), perceived to be large (South), rural mothers (West), higher socio-economic status (Central) were negatively associated.<br><br>Higher maternal education (Central), scheduled tribe (East, North-East), Caesarian delivery (North-East), currently married (North East), frequent ANC visits (≥4) (North) were positively associated.	data collection had high response rates (from 94.0 to 99.6% across the states of India) reducing the potential effect of selection bias. Second, the India DHS data were collected by skilled personnel using standardised questionnaires which ensured that the data collected were consistent across the states and territories of India. Lastly, the study provided evidence on important modifiable factors associated with EBF in the world's second largest populations to help nutrition experts in the country advocate for effective policies and intervention services to improve EBF in India.	A temporal relation could not be established due to the cross-sectional nature of the study. There could be some recall bias due to the self-reporting. There could also be a measurement bias due to the over-reporting or under-reporting of the factors. All the confounding factors were not considered when conducting the study.	9
Chhetri et al.; 2018 [10]	137 working mothers; 0–6 months	Udupi taluk, Karnataka	community based cross-sectional study	High maternal and paternal education, place of delivery (private hospital), female child, frequency of breastfeeding per day, practice of expressing and storing breastmilk before leaving for work and breaks	A validated , pre-designed questionnaire was used.	A temporal relation could not be established due to the cross-sectional nature of the study. Recall bias may have influenced the outcomes.	7

				during working hours were found to be positively associated.			
Nishimura et al.; 2018 [11]	1292 mothers; 0–12 months	Mysore, Karnataka	Cross sectional study	higher/increasing maternal age, lower maternal education, and frequent ANC visits (7–10) were positively associated.	The large sample size and low loss to follow up rate confers greater statistical power and generalizability. The questionnaire was based on validated items from the India's National Family Health Survey-3	Recall bias could be there due to the nature of the interviews. There could be some confounding factors which could influence the outcomes of the study. Additionally, the results are not generalisable to the national population.	10
Velusamy et al.; 2017 [12]	1088 mothers; 0–6 months	Vellore, South India	community based prospective birth cohort study (combining data from 3 studies)	High maternal education, pucca type of house, two or more number of children in the family, nuclear family structure and birth during summer were negatively associated.	Prospective design of the study was major strength. Further, pooling data from three similar birth cohort studies resulted in larger sample size of 1, 088, reducing the risk of chance findings and adding statistical power to the analyses of relevant determinants. Rigorous follow-up allows, information for most children to be available and hence reducing the bias due to attrition.	study was not designed to assess the determinants of exclusive breastfeeding. Additional factors needed to be assessed for key factors factors associated with exclusive breastfeeding would likely include additional relevant factors that were not collected as a part of the existing studies. Also, missing information on antenatal visits, prelacteal feeding, time of initiation of breastfeeding, maternal nutrition, and vaccination schedule did not allow the investigators to investigate these factors.	10
Veeranki et al.; 2017 [13]	1294 mother-infant pairs; 0–12 months	Mysore, Karnataka	prospective cohort study	Older maternal age was negatively associated with nonexclusive breastfeeding. High maternal education was positively associated with non-EBF.	Strong design of prospective cohort and large sample size, which allowed for examining sociodemographic and delivery characteristics associated with strong statistical power and analysis and minimal recall bias.	The findings are not generalisable to the national population of India. There could have been recall bias in the study. Also, there have been other confounding factors such as breastfeeding problems of mothers, previous reproductive history (eg, number of abortions and neonatal deaths), feeding preference of family members, and feeding practices of friends which were not considered for the study and could have influenced the outcomes.	8
Oakley et al.; 2017 [14]	7848 children; <6 year of age	Ranga Reddy district, southern India	Cross sectional study	High maternal education, higher socioeconomic status was positively associated with early termination of EBF.	Validated questionnaire was used. Advanced statistical methods were employed to run the analysis.	Temporal association between outcome and the study factors may not be established due to the nature of the study. There is a possibility of recall bias due to the nature of data collection. There could have been differential misclassification bias due to the nature of the factors.	8
Das et al.; 2016 [17]	20793 mothers of 0–5-month-old children and 10130 mothers of 6–8-month-old children. 0–12months	Bihar	Cross-sectional study	Winter nursing and breastfeeding counselling were positively associated	The large sample size allowed for robust analysis for multiple covariates simultaneously in the regression analyses and to perform age subgroup analyses. Moreover, as the LQAS surveys were conducted across multiple rounds during different times of the year, therefore analyses of the seasonal trends without being concerned about the sample size was possible. Additionally, a uniform protocol and rigorous training methodology was implemented across the survey regions and rounds, which reduced the between-interviewer variations and improved the quality of collected data.	Temporal relation could not be established due to the cross-sectional nature of the study. The study findings are not generalisable to the national population. There could also have been social desirability bias, measurement bias and the recall bias due to the nature of the study and the data collection.	8
Gupta et al.; 2015 [20]	194 mother- children pairs; 0–23 months	Delhi	Community based cross-sectional study	lower birth order, institutional delivery, normal vaginal delivery was positively associated.	A pre-validated standard questionnaire was used	The study findings are not representative of national Indian population. The temporality could not be established due to the cross-sectional nature of the study. There could have been recall bias and mis classification bias in the study.	7
Chandiok et al.; 2015 [21]	34,176 and 25,459 births in NFHS-1 and NFHS-3 respectively; 0–5 months	India	community-based cross-sectional study	In the NFHS-1 infants perceived to be small size at birth and employed mothers were positively associated. While urban residence, younger maternal age (< 20 years), high maternal education, higher SLI	use of validated questionnaire and nationally representative data set over two time points, very high survey response rates, low rates of missing and excluded data and appropriate	Causality can not be established due to the cross-sectional nature of the study. There could be recall bias due to the data collection methods, and there	8

				status, preceding birth interval (< 2 years), ANC care, were negatively associated.<br><br>However, in the NFHS-3, rural residence, low maternal education, employed mothers, ANC care were negatively associated.	adjustments for sampling design made in the analysis.	could be misclassification error leading to under/overestimation of the results.	
Choudhary et al.; 2015 [22]	1000 mothers; Age of children is not mentioned	postnatal care OPD in a tertiary care center- J.P.Hospital in Bhopal, Madhya Pradesh	Cross-sectional observational study	maternal age (20–25 years), high maternal education, high socioeconomic status, multiparity, and availing ANC services were positively associated.	A predesigned, pretested questionnaire was used	The study findings cannot be generalised to the national Indian population. The causality can not be established due to the cross-sectional nature of the study, recall bias and misclassification bias could also be there.	8
Gogoi et al.; 2015 [23]	136 children; 6–24 months	Dibrugarh, Assam	Cross-sectional study, Mixed method model	Mothers from nuclear family, primiparity, frequent ANC visits (≥ 4) were positively associated.	A predesigned, pretested questionnaire was used	The study findings could not be generalised to the national Indian population. The causality cannot be established due to the cross-sectional nature of the study, recall bias and misclassification bias could also be there.	7
Srivastava et al.; 2014 [25]	1020 mothers; 0–6 week infants	Two public hospitals, Lucknow, Uttar Pradesh	Prospective follow up cohort study	low maternal and paternal education was negatively associated.<br><br>Frequent ANC visits (≥ 3), mothers who had two vaccinations of tetanus toxoid (TT) during the antenatal period, Hindus and non-slum dwellers, medium<br>socioeconomic status was positively associated	A predesigned, pretested questionnaire was used	the study was done in public hospitals among mothers from low socio-economic groups and therefore cannot be generalized for all institutional deliveries.the breastfeeding patterns can differ for home-delivered infants and institution delivered infants – hence, cannot be generalised. The study could also not address the reason behind the low prevalence of exclusive breastfeeding in the study population.	9
Mahmood et al.; 2012 [29]	123 women- infant pairs; 0–12 months	Uttar Pradesh	cross-sectional study	Multivariate logistic regression analysis showed that maternity and newborn care variables had no significant association.	Pretested, pre-validated questionnaire was used.	The temporality cannot be established due to the cross-sectional nature of the study, recall bias and misclassification bias could also be there due to the method of data collection. The study findings are not representative of the national population of India.	8
Kumar N. et al.; 2012 [30]	152 infants and mothers	Kasturba Medical College, Mangalore; in Coastal South India	cross-sectional study	Middle to high socioeconomic status were positively associated.	Pretested, pre-validated questionnaire was used.	The temporality cannot be established due to the cross-sectional nature of the study, recall bias and misclassification bias could also be there due to the method of data collection. The study findings are not representative of the national population of India.	8
Bagul et al.; 2021 [31]	384 mother-children pairs	urban slum of Nagpur, Maharashtra	community based, cross-sectional study	High maternal education and breastfeeding counselling by health personals was positively associated.	Pretested, pre-validated questionnaire was used.	The temporality cannot be established due to the cross-sectional nature of the study, recall bias and misclassification bias could also be there due to the method of data collection. The study findings are not representative of the national population of India.	7
Radhakrishnan et al.; 2012 [32]	291 children; 6 months–2 years	Attyampatti Panchyat Union, Salem district, Tamil Nadu	cross-sectional study	Normal vaginal delivery, nuclear family, number of children (< 2), smaller family size (< 4) was positively associated	Pretested, pre-validated questionnaire was used.	The temporality cannot be established due to the cross-sectional nature of the study, recall bias and misclassification bias could also be there due to the method of data collection. The study findings are not representative of the national population of India.	8
Bhanderi et al.; 2011 [33]	300 children under 5 years of age	Petlad town, a semiurban area of Anand district, Gujarat, India	community based, cross-sectional study	Maternal age (22–26 years) was positively associated	Pretested, pre-validated questionnaire was used.	The temporality cannot be established due to the cross-sectional nature of the study, recall bias and misclassification bias could also be there due to the method of data collection. The study findings are not representative of the national population of India.	8
Patel et al.; 2010 [35]	20,108 children; 0–23 months	India	cross-sectional study	High socioeconomic status and health facility delivery were negatively associated.<br><br>Normal vaginal or assisted delivery were positively associated.	Pretested, pre-validated questionnaire was used. A larger sample size was used and it was nationally representative. The findings were generalisable to the Indian population.	The temporality cannot be established due to the cross-sectional nature of the study, recall bias and misclassification bias could also be there due to the method of data collection	9

				As compared with the Northern region, all other regions had higher prevalence.			
Kunwar et al.; 2010 [36]	272 mothers; 6–8 months	Lucknow, Northern India	cross-sectional hospital-based survey	High maternal education was positively associated	A pre-validated, pre-tested questionnaire was used.	The temporality cannot be established due to the cross-sectional nature of the study, recall bias and misclassification bias could also be there due to the method of data collection. The study findings are not generalisable to national population of India.	7
Tiwari et al.; 2009 [38]	279 mother -infants; 6–11 months	Gwalior, India	community-based cross-sectional study	Preterm infants, normal birth weight infants, EIBF, ANC visits (≥3), high maternal education and immunization visits were positively associated.	A pre-validated, pre-tested questionnaire was used.	The temporality cannot be established due to the cross-sectional nature of the study, recall bias and misclassification bias could also be there due to the method of data collection. The study findings are not generalisable to national population of India.	8
Kishore et al.; 2009 [39]	77 mother infant pairs; 0–6 months	Haryana	community-based cross-sectional study	breastfeeding counselling was positively associated	A pre-validated, pre-tested questionnaire was used.	The temporality cannot be established due to the cross-sectional nature of the study, recall bias and misclassification bias could also be there due to the method of data collection. The study findings are not generalisable to national population of India.	9
Chudasama et al.; 2009 [40]	498 infants; 0–12 months	South Gujarat	Cross-sectional study	factors associated with non-EBF/early weaning were primiparity, consecutive delivery interval (<24 months), maternal age (< 20 years), and paternal occupation as labor	A pre-validated, pre-tested questionnaire was used.	The temporality cannot be established due to the cross-sectional nature of the study, recall bias and misclassification bias could also be there due to the method of data collection. The study findings are not generalisable to national population of India.	7

Supplementary Table S5. Continued breastfeeding at 1 year

Author; Year	Number of children/number of mothers/Age of children	Geographical region	Study design	Factors associated with Continued breastfeeding at 1 year	Study Strengths	Study limitations	Quality assessment score
Kumar N. et al.; 2012 [30]	152 infants and mothers	Kasturba Medical College, Mangalore; in Coastal South India	cross-sectional study	Maternal age (21–30 years) and joint family mothers were positively associated	Pretested, pre-validated questionnaire was used.	The temporality cannot be established due to the cross-sectional nature of the study, recall bias and misclassification bias could also be there due to the method of data collection. The study findings are not representative of the national population of India.	8

Supplementary Table S6. Continued breastfeeding at 2 years

Author; Year	Number of children/number of mothers/Age of children	Geographical region	Study design	Factors associated with Continued breastfeeding at 2 years	Study Strengths	Study limitations	Quality assessment score
Oakley et al.; 2017 [14]	7848 children; <6 year of age	Ranga Reddy district, southern India	Cross sectional study	High maternal education, increasing urbanicity were positively associated with breastfeeding discontinuation before 24 months	Validated questionnaire was used. Advanced statistical methods were employed to run the analysis.	Temporal association between outcome and the study factors may not be established due to the nature of the study. There is a possibility of recall bias due to the nature of data collection. There could have been differential misclassification bias due to the nature of the factors.	8
Mehta et al.; 2017 [15]	7534 women	India	cross-sectional	Male children, rural women, younger maternal age at marriage (17 years), delivery assistance by a friend were positively associated.	Response rate was high in the participants. Validated questionnaire was used for the analysis.	Recall bias may have influenced the results. The temporality could not be established due to cross-sectional study. There could have been social desirability bias due to women feeling the pressure of answering in a certain way. Also, there could be other factors like women's occupation which could be influencing the results.	8
Malhotra et al.; 2008 [41]	31645 children; 0–24 months	India	Cross-sectional study	Muslims, Sikhs and Christians, OBCs, increasing maternal education, higher SLI, private hospital deliveries positively associated<br><br>male child, rural residence, increasing maternal age at childbirth, higher birth order, ANC care negatively associated	A pre-validated, pre-tested questionnaire was used. Sample size was nationally representative.	The temporality cannot be established due to the cross-sectional nature of the study, recall bias and misclassification bias could also be there due to the method of data collection. The study findings are not generalisable to national population of India.	9

Supplementary Table S7. Predominant breastfeeding

Author; Year	Number of children/number of mothers/ Age of children	Geographical region	Study design	Factors associated with Predominant breastfeeding	Study Strengths	Study limitations	Quality assessment score
Srivastava et al.; 2014 [25]	1020 mothers; 0–6 week infants	Two public hospitals, Lucknow, Uttar Pradesh	Prospective follow up cohort study	low maternal and paternal education, fewer (<3) ANC visits, had fewer TT vaccinations, Muslims, slum dwellers, lower socioeconomic status were positively associated.	A predesigned, pretested questionnaire was used	the study was done in public hospitals among mothers from low socio-economic groups and therefore cannot be generalized for all institutional deliveries.the breastfeeding patterns can differ for home-delivered infants and institution delivered infants – hence, cannot be generalised. The study could also not address the reason behind the low prevalence of exclusive breastfeeding in the study population.	9

Supplementary Table S8. Bottle feeding

Author; Year	Number of children/number of mothers/Age of children	Geographical region	Study design	Factors associated with Bottle feeding	Study Strengths	Study limitations	Quality assessment score
Patel et al.; 2010 [35]	20,108 children; 0–23 months	India	cross-sectional study	Smaller babies and those born without the assistance of a health professional were negatively associated.<br><br>Employed mothers, high maternal education, high socioeconomic status, urban residence, and those watching television, had higher prevalence.<br><br>As compared with the Northern region, the Central region had higher prevaalence, whereas the North-Eastern and Western regions had lower prevalence	Pretested, pre-validated questionnaire was used. A larger sample size was used and it was nationally representative. The findings were generalisable to the Indian population.	The temporality cannot be established due to the cross-sectional nature of the study, recall bias and misclassification bias could also be there due to the method of data collection	9

Supplementary Table S9. Introduction to solid, semi-solid or soft foods

Author; Year	Number of children/number of mothers/Age of children	Geographical region	Study design	Factors associated with Introduction to solid, semi-solid or soft foods	Study Strengths	Study limitations	Quality assessment score
Dhami et al.; 2019 [8]	69,464; 6–23 months children	India	Cross-sectional study	higher socio-economic status (North, East), urban women (West), higher birth order (Central), frequent ANC visits (≥4) (Eastern and Central) were positively associated.	The study used the most recent and nationally representative data (NFHS-4) for India. The NFHS-4 data were obtained from a larger sample compared to previous national surveys, indicating that findings are more generalisable to the Indian population. The data used are comparable across regions in India given that they were collected by trained personnel who used standardized questionnaires and methodology. The study findings are unlikely to be affected by selection bias as the survey yielded high responses rates, over 94%.	A temporal relation could not be established due to the cross-sectional nature of the study. There could be some recall bias due to the self-reporting. There could also be a measurement bias due to the over-reporting or under-reporting of the factors. All the confounding factors were not considered when conducting the study.	9
Kakati et al.; 2016 [19]	250 infants; 7–12 months	Kamrup district, Assam, India	Community based cross-sectional study	The infants born at Government. Institution, high socio-economic status, high maternal education, normal delivery, higher parity was positively associated.	A pre-validated standard questionnaire was used	The study findings are not generalisable to the national population of India. The causality could not be established due to the cross-sectional nature of the study. There could have been recall bias and information bias in the study.	8
Gupta et al.; 2015 [20]	194 mother- children pairs; 0–23 months	Delhi	Community based cross-sectional study	higher maternal education and male child were positively associated.	A pre-validated standard questionnaire was used	The study findings are not representative of national Indian population. The temporality could not be established due to the cross-sectional nature of the study. There could have been recall bias and mis classification bias in the study.	7
Malhotra et al.; 2013 [27]	9241 children aged; 6–18 months	India	Cross-sectional community-based study	ANC visits, health professional advise were positively associated	Pretested, pre-validated questionnaire was used.	The temporality cannot be established due to the cross-sectional nature of the study, recall bias and misclassification bias could also be there.	8
Patel et al.; 2012 [28]	15,028 last-born children; 6–23 months	India	cross-sectional study	High socio-economic status, ≥ 6 ANC visits, mothers reading newspaper were positively associated. South, North East residents were positively associated.	The ability to determine the most susceptible age group and the modifiable factors that affect inappropriate practices in a large sample size which allows for control of confounders. The sample is nationally representative. A pre-validated questionnaire was used.	The temporality cannot be established due to the cross-sectional nature of the study, recall bias and misclassification bias could also be there due to the method of data collection.	9
Bhanderi et al.; 2011 [33]	300 children under 5 years of age	Petlad town, a semiurban area of Anand district, Gujarat, India	community based, cross-sectional study	High maternal education, ANC care were positively associated	Pretested, pre-validated questionnaire was used.	The temporality cannot be established due to the cross-sectional nature of the study, recall bias and misclassification bias could also be there due to the method of data collection. The study findings are not representative of the national population of India.	8
Rao et al.; 2011 [34]	200 mothers of children; aged 6–24 months	Dr TMA Pai Hospital Udupi and Dr TMA Pai Hospital Karkala and a public hospital, Regional Advanced Paediatric Care Centre, Mangalore.	Hospital based cross-sectional study	Middle socioeconomic status, higher birth order, hospital delivery, higher maternal education positively associated.	Pretested, pre-validated questionnaire was used.	The temporality cannot be established due to the cross-sectional nature of the study, recall bias and misclassification bias could also be there due to the method of data collection. The study findings are not representative of the national population of India. Feed consistency was not taken into consideration for complementary feeding practices. Some of the questions asked were not open-ended. Additionally, the timescale over which the study was conducted was also a limitation.	7
Patel et al.; 2010 [35]	20,108 children; 0–23 months	India	cross-sectional study	The prevalence was higher for women with frequent ANC visits (≥7) and for those who watched television.<br><br>The prevalence was higher in the Southern, North-Eastern, and Eastern regions than in the Northern region.	Pretested, pre-validated questionnaire was used. A larger sample size was used and it was nationally representative. The findings were generalisable to the Indian population.	The temporality cannot be established due to the cross-sectional nature of the study, recall bias and misclassification bias could also be there due to the method of data collection	9
Jayant et al.; 2010 [37]	300 children; 0–5 years	Pravara Rural Hospital, Loni, Maharastra	cross-sectional descriptive study	High maternal education was positively associated.	A pre-validated, pre-tested questionnaire was used.	The temporality cannot be established due to the cross-sectional nature of the study, recall bias and misclassification bias could also be there due to the method of data collection.	7

		The study findings are not generalisable to national population of India.

Supplementary Table S10. Minimum Dietary Diversity

Author; Year	Number of children/number of mothers/Age of children	Geographical region	Study design	Factors associated with Minimum Dietary Diversity	Study Strengths	Study limitations	Quality assessment score
Dhami et al.; 2019 [8]	69,464; 6–23 months children	India	Cross-sectional study	higher socio-economic status (North, West, Central, North East), higher maternal education (North, Central), woman autonomy over power of earnings (South), higher birth order (North, South, East, West, North East), frequent ANC visits (≥4) (East) were positively associated.	The study used the most recent and nationally representative data (NFHS-4) for India. The NFHS-4 data were obtained from a larger sample compared to previous national surveys, indicating that findings are more generalisable to the Indian population. The data used are comparable across regions in India given that they were collected by trained personnel who used standardized questionnaires and methodology. The study findings are unlikely to be affected by selection bias as the survey yielded high responses rates, over 94%.	A temporal relation could not be established due to the cross-sectional nature of the study. There could be some recall bias due to the self-reporting. There could also be a measurement bias due to the over-reporting or under-reporting of the factors. All the confounding factors were not considered when conducting the study.	9
Ahmad et al.; 2017 [16]	326 children; 6–23 months	Jawaharlal Nehru Medical College, Aligarh Muslim University, Aligarh.	community-based, cross-sectional study	urban residence, high birth order and High Standard of living index (SLI) was positively associated	A pre-validated standard questionnaire was used.	The study findings represent only a small section of the community and are not generalisable. There could have been recall bias. Also, the causality can not be established due to the cross-sectional nature of the study.	8
Malhotra et al.; 2013 [27]	9241 children aged; 6–18 months	India	Cross-sectional community-based study	Media exposure to radio, reading newspaper was positively associated	Pretested, pre-validated questionnaire was used.	The temporality cannot be established due to the cross-sectional nature of the study, recall bias and misclassification bias could also be there.	8
Patel et al.; 2012 [28]	15,028 last-born children; 6–23 months	India	cross-sectional study	Low socioeconomic status, low maternal education, lower exposure to media (radio, television or newspaper), fewer (< 6 to none) ANC visits were negatively associated. East was negatively associated.	The ability to determine the most susceptible age group and the modifiable factors that affect inappropriate practices in a large sample size which allows for control of confounders. The sample is nationally representative. A pre-validated questionnaire was used.	The temporality cannot be established due to the cross-sectional nature of the study, recall bias and misclassification bias could also be there due to the method of data collection.	9

Supplementary Table S11. Minimum Meal Frequency

Author; Year	Number of children/number of mothers/Age of children	Geographical region	Study design	Factors associated with Minimum Meal Frequency	Study Strengths	Study limitations	Quality assessment score
Dhami et al.; 2019 [8]	69,464; 6–23 months children	India	Cross-sectional study	higher socio-economic status (South, East), higher maternal education (North, South, Central), woman autonomy over finances (Central), higher birth order (North, East, Central, North East), TBA- and health professional-assisted births (East), frequent ANC visits (≥4) (North, South, East, Central) were positively associated	The study used the most recent and nationally representative data (NFHS-4) for India. The NFHS-4 data were obtained from a larger sample compared to previous national surveys, indicating that findings are more generalisable to the Indian population. The data used are comparable across regions in India given that they were collected by trained personnel who used standardized questionnaires and methodology. The study findings are unlikely to be affected by selection bias as the survey yielded high responses rates, over 94%.	A temporal relation could not be established due to the cross-sectional nature of the study. There could be some recall bias due to the self-reporting. There could also be a measurement bias due to the over-reporting or under-reporting of the factors. All the confounding factors were not considered when conducting the study.	9
Ahmad et al.; 2017 [16]	326 children; 6–23 months	Jawaharlal Nehru Medical College, Aligarh Muslim University, Aligarh.	community-based, cross-sectional study	urban residence, male child, higher maternal education was positively associated.	A pre-validated standard questionnaire was used.	The study findings represent only a small section of the community and are not generalisable. There could have been recall bias. Also, the causality can not be established due to the cross-sectional nature of the study.	8
Malhotra et al.; 2013 [27]	9241 children aged; 6–18 months	India	Cross-sectional community-based study	Media exposure to radio, reading newspaper was positively associated	Pretested, pre-validated questionnaire was used.	The temporality cannot be established due to the cross-sectional nature of the study, recall bias and misclassification bias could also be there.	8
Patel et al.; 2012 [28]	15,028 last-born children; 6–23 months	India	cross-sectional study	Women with child of 6–17 months, low education, did not read newspaper, less power in household decision making, less frequent ANC visits (< 6 to none), lower socio-economic status were negatively associated. West, and North were negatively associated.	The ability to determine the most susceptible age group and the modifiable factors that affect inappropriate practices in a large sample size which allows for control of confounders. The sample is nationally representative. A pre-validated questionnaire was used.	The temporality cannot be established due to the cross-sectional nature of the study, recall bias and misclassification bias could also be there due to the method of data collection.	9

Supplementary Table S12. Minimum Acceptable Diet

Author; Year	Number of children/number of mothers/Age of children	Geographical region	Study design	Factors associated with Minimum Acceptable Diet	Study Strengths	Study limitations	Quality assessment score
Dhami et al.; 2019 [8]	69,464; 6–23 months children	India	Cross-sectional study	higher socio-economic status (North, South), maternal age (≥ 25 years) (South), higher birth order (North, East, Central, North-East), health facility delivery (South), frequent ANC visits (≥4) (East, Central) were positively associated	The study used the most recent and nationally representative data (NFHS-4) for India. The NFHS-4 data were obtained from a larger sample compared to previous national surveys, indicating that findings are more generalisable to the Indian population. The data used are comparable across regions in India given that they were collected by trained personnel who used standardized questionnaires and methodology. The study findings are unlikely to be affected by selection bias as the survey yielded high responses rates, over 94%.	A temporal relation could not be established due to the cross-sectional nature of the study. There could be some recall bias due to the self-reporting. There could also be a measurement bias due to the over-reporting or under-reporting of the factors. All the confounding factors were not considered when conducting the study.	9
Ahmad et al.; 2017 [16]	326 children; 6–23 months	Jawaharlal Nehru Medical College, Aligarh Muslim University, Aligarh.	community-based, cross-sectional study	urban residence, male child, higher birth order, higher SLI was positively associated.	A pre-validated standard questionnaire was used.	The study findings represent only a small section of the community and are not generalisable. There could have been recall bias. Also, the causality can not be established due to the cross-sectional nature of the study.	8
Patel et al.; 2012 [28]	15,028 last-born children; 6–23 months	India	cross-sectional study	Women with child of 6–17 months, low education, did not read newspaper, less power in household decision making, less frequent ANC visits (< 6 to none), lower socio-economic status were negatively associated. East was negatively associated.	The ability to determine the most susceptible age group and the modifiable factors that affect inappropriate practices in a large sample size which allows for control of confounders.<br><br>The sample is nationally representative. A pre-validated questionnaire was used.	The temporality cannot be established due to the cross-sectional nature of the study, recall bias and misclassification bias could also be there due to the method of data collection.	9
Rao et al.; 2011 [34]	200 mothers of children aged 6–24 months	Dr TMA Pai Hospital Udupi and Dr TMA Pai Hospital Karkala and a public hospital, Regional Advanced Paediatric Care Centre, Mangalore.	Hospital based cross-sectional study	Hospital delivery was positively associated	Pretested, pre-validated questionnaire was used.	The temporality cannot be established due to the cross-sectional nature of the study, recall bias and misclassification bias could also be there due to the method of data collection. The study findings are not representative of the national population of India. Feed consistency was not taken into consideration for complementary feeding practices. Some of the questions asked were not open-ended. Additionally, the timescale over which the study was conducted was also a limitation.	7
